# Stress induced nuclear granules form in response to accumulation of misfolded proteins in *Caenorhabditis elegans*

**DOI:** 10.1186/s12860-017-0136-x

**Published:** 2017-04-19

**Authors:** Katherine M. Sampuda, Mason Riley, Lynn Boyd

**Affiliations:** Department of Biology, Middle Tennessee State University, 1301 E. Main Street, Murfreesboro, TN 37132 USA

**Keywords:** Ubiquitin, Proteasome, Nuclear body, Salt stress, Oxidative stress, Starvation

## Abstract

**Background:**

Environmental stress can affect the viability or fecundity of an organism. Environmental stressors may affect the genome or the proteome and can cause cellular distress by contributing to protein damage or misfolding. This study examines the cellular response to environmental stress in the germline of the nematode, *C. elegans*.

**Results:**

Salt stress, oxidative stress, and starvation, but not heat shock, induce the relocalization of ubiquitin, proteasome, and the TIAR-2 protein into distinct subnuclear regions referred to as stress induced nuclear granules (SINGs). The SINGs form within 1 h of stress initiation and do not require intertissue signaling. K48-linked polyubiquitin chains but not K63 chains are enriched in SINGs. Worms with a mutation in the conjugating enzyme, *ubc-18*, do not form SINGs. Additionally, knockdown of *ubc-20* and *ubc-22* reduces the level of SING formation as does knockdown of the ubiquitin ligase *chn-1,* a CHIP homolog. The nuclear import machinery is required for SING formation. Stressed embryos containing SINGs fail to hatch and cell division in these embryos is halted. The formation of SINGs can be prevented by pre-exposure to a brief period of heat shock before stress exposure. Heat shock inhibition of SINGs is dependent upon the HSF-1 transcription factor.

**Conclusions:**

The heat shock results suggest that chaperone expression can prevent SING formation and that the accumulation of damaged or misfolded proteins is a necessary precursor to SING formation. Thus, SINGs may be part of a novel protein quality control system. The data suggest an interesting model where SINGs represent sites of localized protein degradation for nuclear or cytosolic proteins. Thus, the physiological impacts of environmental stress may begin at the cellular level with the formation of stress induced nuclear granules.

**Electronic supplementary material:**

The online version of this article (doi:10.1186/s12860-017-0136-x) contains supplementary material, which is available to authorized users.

## Background

Organisms are faced with a variety of environmental conditions, some of which can adversely affect the status of proteins. Cells have multiple systems that monitor and maintain the proteome. Protein quality control systems help to refold proteins, to sequester proteins, or to degrade damaged or misfolded proteins. The ubiquitin proteasome system (UPS) is the major intracellular protein degradation pathway. This system is essential for removing damaged or misfolded proteins [1, 2]. In the current study, we investigate how the UPS is involved in the response to environmental stressors in the germline of the nematode, *Caenorhabditis elegans.* Ubiquitin is an 8.5 kDa polypeptide. Three distinct enzymatic activities link ubiquitin to the substrate protein via an isopeptide bond between the C-terminal glycine of ubiquitin and the amino group on a lysine residue of the substrate. This process is used to either add a single ubiquitin or a polyubiquitin chain. Different types of polyubiquitin chains form depending on the lysine linkage used. K48 polyubiquitin chains are recognized by the proteasome [3] and K63 chains are associated with protein trafficking, NFκB activation, and receptor endocytosis [4, 5].

Protein quality control systems exist for proteins in the cytosol, the mitochondria, and the endoplasmic reticulum [6]. However, the control of protein quality in the nucleus is not well understood. Ubiquitin and proteasome are both found in the nucleus along with various chaperones [7]. Proteasome activity has been detected in the nuclei of mammalian cells [8]. Therefore, the machinery needed for protein quality control exists in the nucleus, but details on the pathway for triggering nuclear protein degradation is not known. The best described examples of nuclear protein degradation come from yeast where the San1p ubiquitin ligase is known to target unstable proteins for nuclear degradation [9]. Also in yeast, misfolded cytoplasmic proteins can be imported into the nucleus for degradation [10]. It is not presently clear if this same type of pathway exists in other organisms.

There are several documented nuclear changes in response to stress. The nuclei of cells in various model organisms are known to develop distinct nuclear bodies [11, 12]. These nuclear bodies often vary in size, lack a defining membrane, and are spherical in shape. Nuclear bodies that form in response to stress include promyelocytic leukemia bodies (PML), heat-shock bodies, paraspeckles, clastosomes, nucleoplasmic speckles, and insulator bodies [13–16]. Heat-shock bodies form as a result of elevated temperatures, which leads to the activation of HSF1 [14, 17]. PML bodies form in response to elevated levels of oxidative stress and increase in numbers and size as stress exposure is extended [18–20]. Osmotic stress also induces formation of clastosomes and insulator bodies [15, 16]. Some nuclear bodies are known to contain ubiquitin and proteasome components [21]. Clastosomes contain both ubiquitin conjugates and 19S and 20S proteasome complexes, and disappear when proteasome inhibitors are added. These nuclear bodies are proposed to be active sites of proteolysis [15]. Proteasome components have also been observed in nucleoplasmic speckles and PML nuclear bodies [22–24]. Currently, there is a poor understanding of nuclear bodies’ physiological role and how they are connected to the cellular stress response.

Previous studies in *C. elegans* have shown that exposure to hypertonic stress increases the level of ubiquitin conjugates [25]. That study also showed that expression of the proteasome was required for worms to survive threshold levels of hypertonic stress indicating that protein quality control is a critical aspect of surviving environmental stress. The current study further elaborates on the role of the UPS in response to environmental stress in the germline of *C. elegans.* We show that ubiquitin and the proteasome respond to salt stress, oxidative stress, and starvation by forming Stress Induced Nuclear Granules (SINGs). These SINGs form quickly after stress is initiated and do not require intertissue signaling. The SINGs are enriched in K48 polyubiquitin chains suggesting that they may be sites of protein degradation. The expression of chaperones reduces the frequency of SINGs suggesting a model where the accumulation of misfolded proteins leads to SING formation.

## Results

### Stress induces foci with K48 polyubiquitin chains and proteasomes in the nucleus

**Fig. 1:**
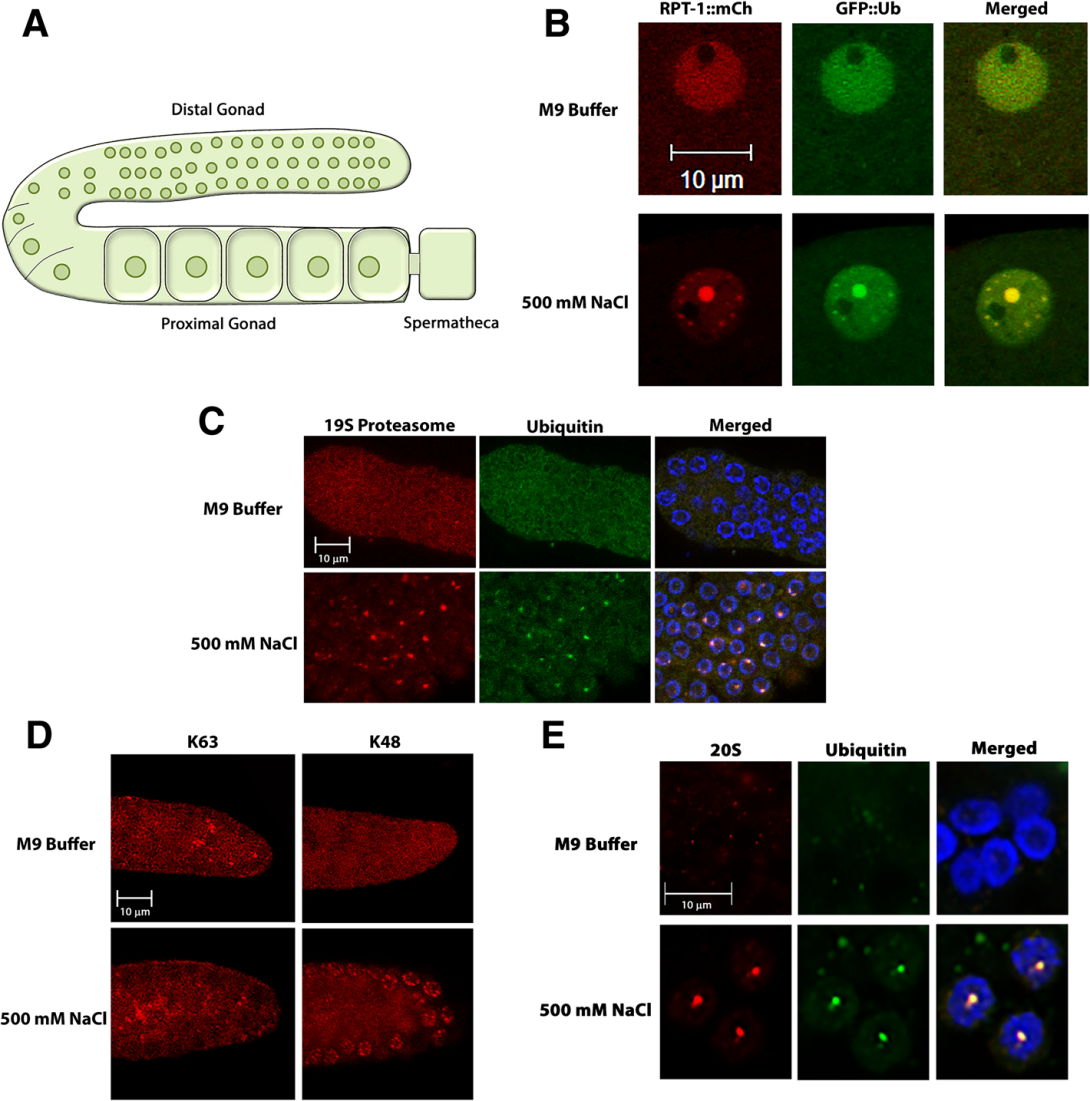
Salt stress induces redistribution of ubiquitin and proteasome into nuclear stress bodies. **a** Reproductive tissue of *C. elegans.* The *C. elegans* gonad is a U-shaped organ with mitotically dividing cells at the distal tip. The distal region of the gonad is syncytial and oocytes become increasingly cellularized and larger as they progress through the proximal region towards the spermatheca. **b** Live imaging of GFP::Ub (*green*) and RPT-1::mCh (*red*) in proximal oocytes. During unstressed (M9 buffer) conditions ubiquitin and 19S proteasome were present in both nucleus and cytoplasm, but appeared to be more concentrated in the nucleus. The merged image shows ubiquitin, proteasome and DAPI channels. In stressed (500 mM NaCl) conditions ubiquitin and RPT-1 concentrate in stress bodies in the nucleus. A single oocyte is shown in each row. SINGs were found in salt stress (66/80 oocytes), but were absent in unstressed conditions (0/80) oocytes). Data were collected from 2 independent experiments (*n* = 20 worms). Scale bar indicates 10 μm. **c** Antibody staining of ubiquitin and 19S proteasome in the distal gonad. Gonads of unstressed and stressed young adult *C. elegans* were dissected out and stained with an ubiquitin antibody (*green*) and a 19S proteasome antibody (*red*). The merged image shows the ubiquitin, proteasome, and DAPI channels. Both ubiquitin and proteasome where found to be diffuse within the nucleus in unstressed conditions (0/600 oocytes with SINGs), but localized into SINGs during salt stress (468/600 oocytes). Data were collected from 3 independent experiments (*n* = 30 worms). Scale bar indicates 10 μm. **d** K63 and K48 polyubiquitin antibody staining in the distal gonad. During exposure to 500 mM NaCl K48 chains localized into SINGs (552/600 oocytes), whereas, K63 chains show no localization to SINGs in response to stress (0/600 oocytes). Data were collected from 3 independent experiments (*n* = 30 worms). Scale bar indicates 10 μm. **e** 20S proteasome subunit antibody staining. An antibody to the alpha 1 subunit of the 20S proteasome (*green*) was used to stain stressed (500 mM NaCl) and unstressed (M9) gonads. A region of the distal gonad is shown for each. The merged image shows the ubiquitin, proteasome, and DAPI channels. In unstressed conditions, SINGs containing 20S proteasome do not form (10/200 oocytes). Under stressed conditions the 20S proteasome subunit colocalizes with K48 ubiquitin chains (*red*) in SINGs (153/200 oocytes). Data were collected from 3 independent experiments (*n* = 30 worms). Scale bar indicates 10 μm

For this investigation, the germline of *C. elegans* is used as a model for the cellular response to stress in reproductive tissue. We took advantage of existing *C. elegans* strains that express fluorescent fusion proteins in the germline [26]. Green fluorescent protein is fused to the N-terminus of ubiquitin (GFP::Ub) thus preserving its ability to be conjugated onto other proteins. mCherry is fused onto the C-terminus of RPT-1 (RPT-1::mCh), a component of the 19S regulatory cap of the 26S proteasome. To study the subcellular distribution of UPS components during stress, adults expressing GFP::Ub and RPT-1::mCh were soaked in either M9 buffer (86 mM NaCl) or 500 mM NaCl for 60 min, and then imaged under confocal microscopy. In unstressed worms, GFP::Ub and RPT-1::mCh exhibit a diffuse pattern in both the cytoplasm and the nucleus of oocytes (Fig. 1b). Under stress conditions, ubiquitin and proteasome relocalize into stress induced nuclear granules (SINGs). These SINGs initially appear in the distal oocytes and soon after in all oocytes. A range of salt concentrations induce SING formation, but 500 mM NaCl was chosen based on the fact that it induces SINGs after approximately 30 minutes and does not result in fatality of the organism.

To test whether the SINGs are artifacts of the fluorescent protein fusions, we used antibodies specific for ubiquitin, the RPT-3 subunit of the 19S proteasome, and the PAS-6 (alpha 1) subunit of the 20S proteasome. The antibody stains show that both endogenous ubiquitin and proteasome localize to SINGs after exposure to 500 mM NaCl (Fig. 1c and e). This indicates that the fluorescent reporters act as credible indicators of the localization of endogenous ubiquitin and proteasome. As can be seen in the different panels in Fig. 1, the SINGs vary in size and fluorescent intensity. Generally, larger SINGs appear in the proximal oocytes but smaller SINGs exist in the distal region. However, even in proximal oocytes there can be both large SINGs and smaller SINGs as is seen in the example in Fig. 1b.

Exposure to high salt could cause cellular distress via a variety of mechanisms such as creating osmotic stress or by disrupting intermolecular and intramolecular charge-based interactions. In order to distinguish between these two scenarios, we used a non-ionic osmotic stress and looked for SING formation. Worms treated with a high concentration of sucrose also form SINGs (Additional file 1: Figure S1) suggesting that osmotic stress may be the instigator of SING formation. To explore whether SINGs might be sites of protein aggregation that form in response to salt stress, we performed FRAP analysis of SINGs and compared the behavior of GFP::Ub in SINGs to that of Q82::GFP, a polyglutamine containing protein that forms aggregates in the cytoplasm of muscle cells [27]. The GFP::Ub in SINGs shows much greater mobility than that of Q82::GFP in aggregates indicating that SINGs are not likely to be sites of protein aggregation (Additional file 2: Figure S2).

Polyubiquitin chains form via an isopeptide linkage between the C-terminal glycine of one ubiquitin and a lysine side chain of another ubiquitin. These chains can be formed by attachment to one of seven different lysines in ubiquitin. All types of chains have been found in cells but the K48 and K63 chains are the most abundant [28]. In order to examine the linkage specificity of ubiquitin in SINGs, we used anti-K48 and anti-K63 polyubiquitin antibodies. K48-linked chains localize to specific nuclear foci under salt stressed conditions in oocytes but K63 chains do not (Fig. 1d). The presence of K48-linked chains in nuclear foci of stressed individuals along with the presence of proteasome is consistent with a model suggesting that proteolysis may be occurring at SING sites and that these sites may be participating in a protein quality control system.

### SINGs form in response to accumulation of misfolded proteins and require nuclear import

**Fig. 2:**
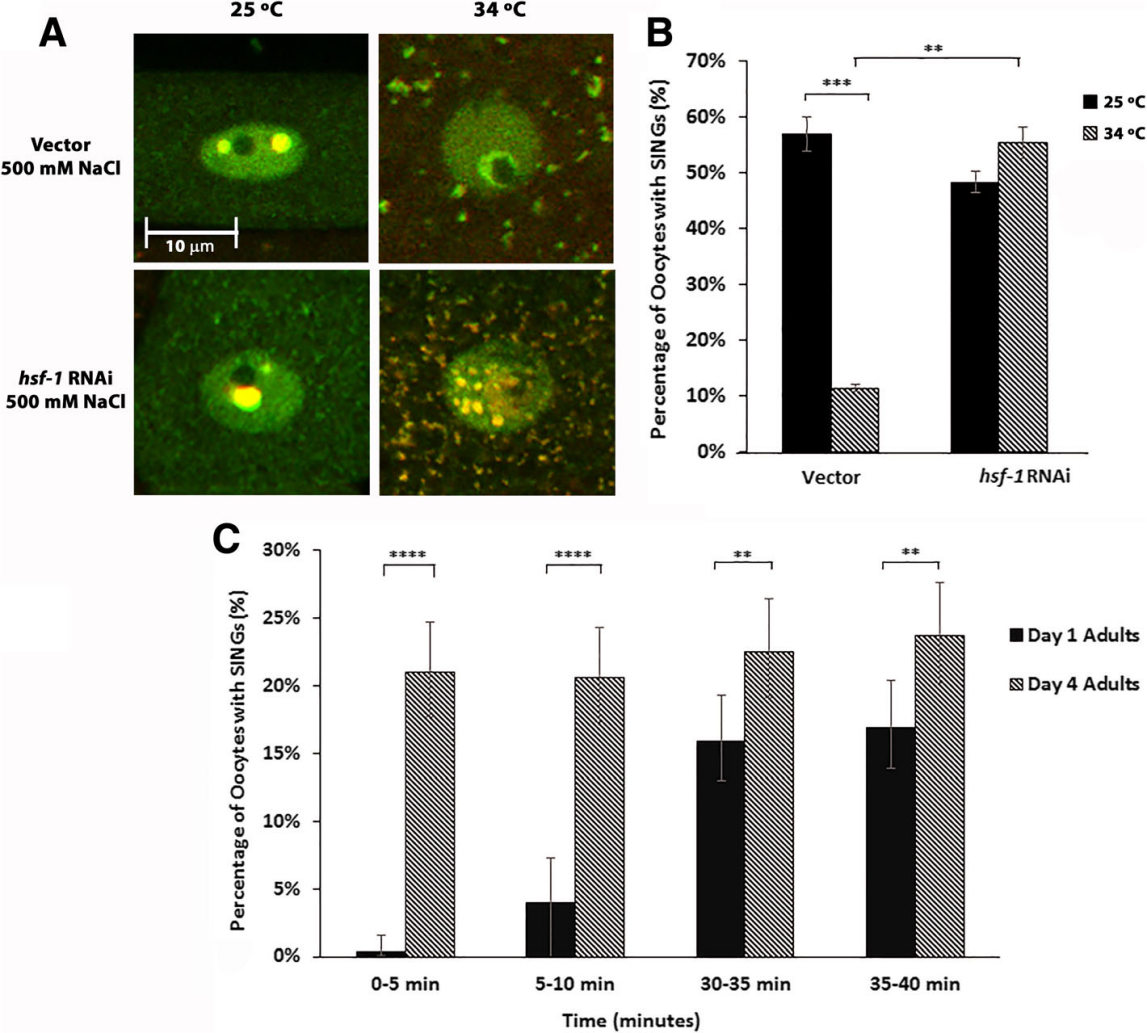
SING formation correlates with reduced protein quality control. **a** Worms expressing GFP::Ub and RPT-1::mCh were incubated for 60 min at either 25 °C or 34 °C prior to soaking in 500 mM NaCl for 60 min. The merged image is shown. Areas that appear yellow have both GFP and mCherry. The brief heat shock reduced the occurrence of SING. Cytoplasmic granules also appear as a result of the heat shock. When *hsf-1* was knocked down via RNAi, SING formation was normal even after heat shock. Scale bar indicates 10 μm. **b** The percentage of oocytes forming SINGs as described in A. A total of 1020 oocytes were observed for each condition from 3 independent experiments (*n* = 30 worms). Statistically relevant differences ***p < 0.0002, two-tailed *z* test. **c** Percentage of oocytes forming SINGs in day 1 and day 4 adults after exposure to 500 mM NaCl for the times indicated. A total of 510 oocytes were observed for each time point from 3 independent experiments (*n* = 30 worms). Statistical significance was calculated by a Fisher’s Exact test: **p < 0.01 and ****p < 0.0001

Since SING formation occurs in response to stress, we hypothesized that SINGs might be a response to protein misfolding and might be involved in protein quality control. Therefore, we tested if chaperone induction via a short heat pulse would affect SING formation. Worms that were incubated at 34 °C for 60 min prior to soaking in 500 mM NaCl did not form SINGs (Fig. 2a and b). Heat shock alone did not induce SING formation (data shown below). HSF-1 is a transcription factor that induces the expression of protein chaperones in response to heat shock. If worms are depleted of HSF-1 via RNAi prior to the 60 min heat shock and subsequent salt stress, then SINGs do form (Fig. 2a and b). This result suggests that expression of chaperones is required for the abrogation of SING formation after heat shock and that SINGs may be involved in a protein quality control pathway.

It is known that protein quality control systems change as an organism ages. In *C. elegans,* chaperone induction declines in aging worms [29] and the amount of protein aggregation increases [30]. When 4 day old worms were subjected to salt stress, SING formation happened more rapidly than in younger adults (Fig. 2c). This result taken together with our finding that prior heat shock can prevent SING formation is consistent with a scenario where cellular stress that causes an increase in protein misfolding also leads to the formation of nuclear granules that contain ubiquitin and proteasome.

**Fig. 3:**
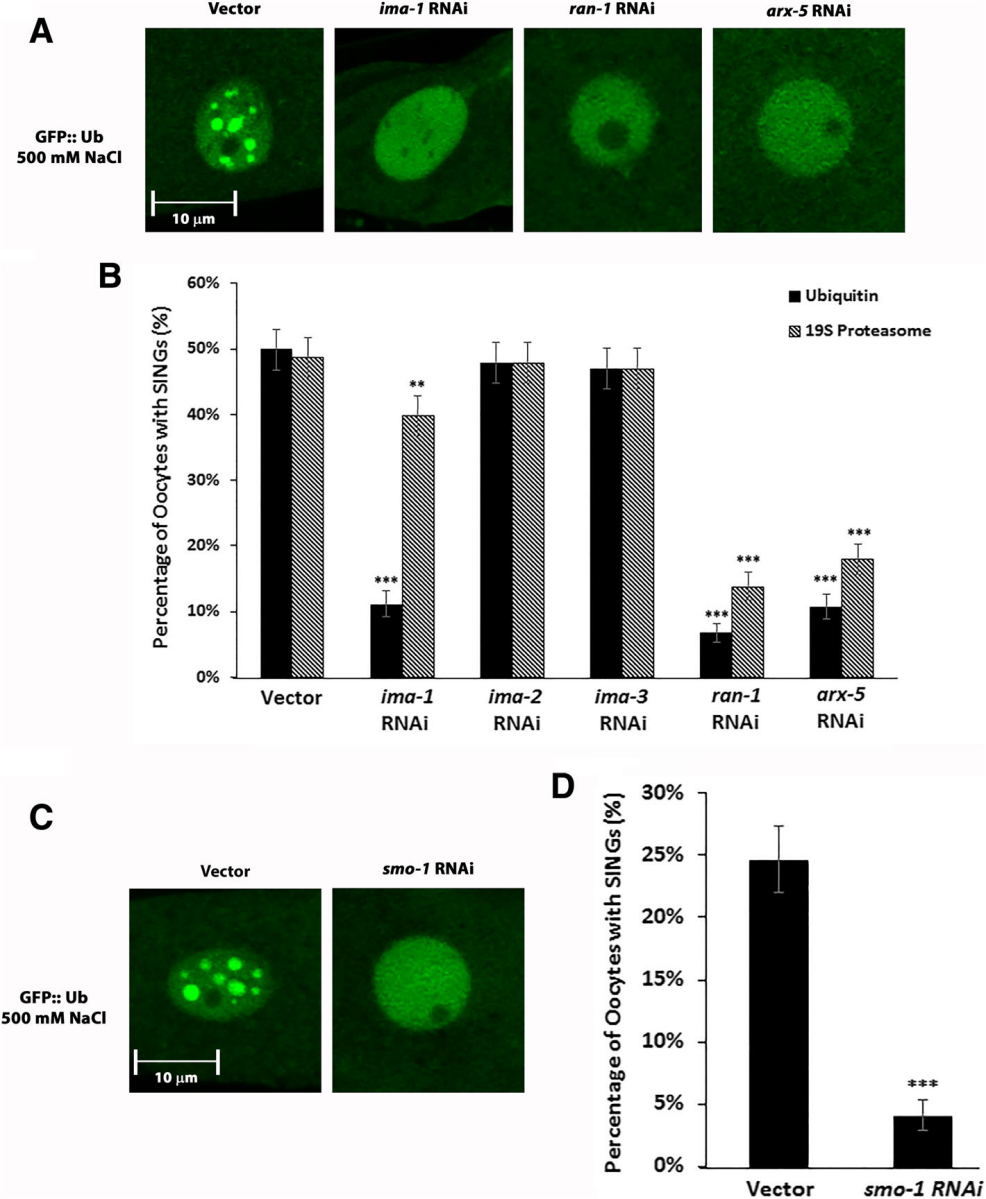
SING formation required the nuclear import pathway. **a** RNAi of *ima-1, ran-1,* and *arx-5* reduces SING formation. Worms expressing GFP::Ub in the germline were soaked in 500 mM NaCl for 60 min. Proximal oocytes are shown. Control worms treated with vector RNAi formed SINGS normally. Scale bar indicates 10 μm. **b** The percentage of oocytes forming SINGs after treatment as described in A. Oocytes were scored for both GFP::Ub and RPT-1::mCh at SINGs. A total of 1020 oocytes were analyzed for each condition from 3 independent experiments (*n* = 30 worms). Statistical significance was calculated by a two-tailed *z* test: **p < 0.01 and ***p < 0.001. **c** RNAi of *smo-1* reduces SING formation in a worm strain expressing GFP::Ub in the germline. Proximal oocytes are shown. Scale bar indicates 10 μm. **d** The percentage of oocytes forming SINGs after treatment as described in C. A total of 1020 oocytes were analyzed from 3 independent experiments (*n* = 30 worms). Statistical significance was calculated by a two-tailed *z* test: ***p < 0.001

The above experiments indicated that protein misfolding is associated with SING formation. In order to investigate whether this is strictly a nuclear event or whether cytoplasmic proteins might be involved, we tested the requirement for nuclear import in this process. RNAi of one of the three importins in *C. elegans* reduced the level of SINGs after stress (Fig. 3a and b). The level of ubiquitin at SINGs was more effected by *ima-1 (RNAi)* than the level of proteasome. This could indicate either that different import pathways are used for ubiquitin and proteasome or that proteasome comes primarily from nuclear stores. However, *arx-5*, a homolog of Arp2/3, did reduce the level of proteasome at SINGs (Fig. 3a and b). Arc3 (an Arp2/3 homolog) is required in yeast for nuclear import of the proteasome [31]. Two other nuclear import components, Ran and SUMO, were also tested. RNAi of Ran (*ran-1)* and SUMO (*smo-1*) reduced SINGs (Fig. 3). Interestingly, SUMO itself does not localize to SINGs (Additional file 3: Figure S5C). The lack of SUMO at SINGs distinguishes them from PML bodies that also form in response to stress but do contain high concentrations of SUMO [19].

Thus, nuclear import is required for SING formation. However, at this time it is not clear whether ubiquitinated substrates and/or proteasomes themselves are imported into the nucleus or whether some other factor such as an E3 ligase that ubiquitinates substrates in the nucleus is being transported into the nucleus.

### The UPS is required for SING formation

**Fig. 4:**
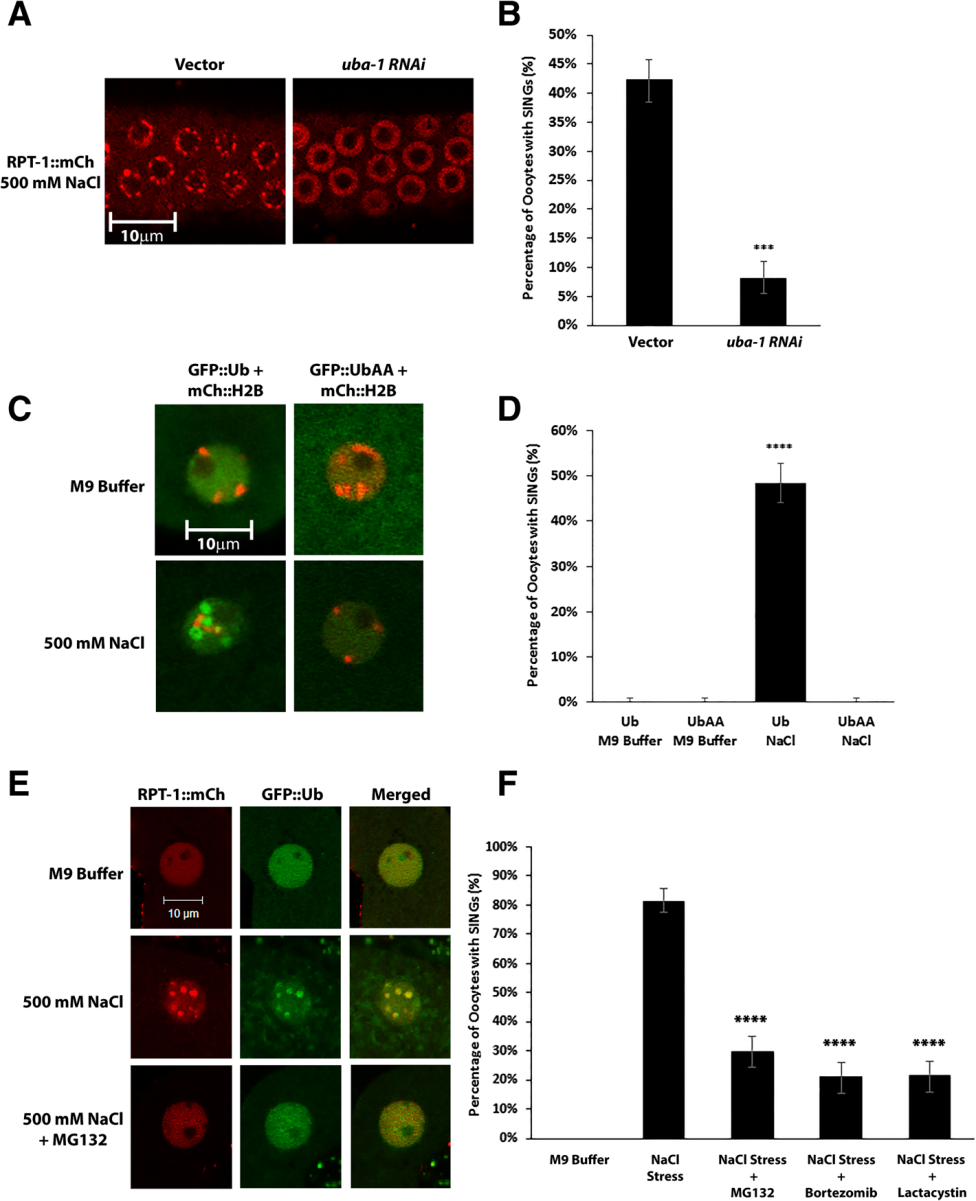
SING formation requires ubiquitination and proteasome activity. **a** Worms expressing RPT-1::mCh were subjected to *uba-1* RNAi for 24 h prior to soaking in 500 mM NaCl for 60 min. RPT-1::mCh does not localize to SINGs when *uba-1* is knocked down. Scale bar indicates 10 μm. **b** The percentage of oocytes forming SINGs after *uba-*1 RNAi treatment as described in A. A total of 1020 oocytes were analyzed from 3 independent experiments (*n* = 30 worms). Statistical significance was calculated by a two-tailed *z* test: ***p < 0.001. **c** Unconjugated ubiquitin does not localize to SINGs. A worm strain expressing GFP::Ub (*green*) and mCh::H2B (*red*) shows localization of GFP::Ub to SINGs after soaking in 500 mM NaCl for 60 min. Whereas, GFP::UbAA does not localize to SINGs. GFP::UbAA lacks a C-terminal diglycine that is required for ubiquitin conjugation. Scale bar indicates 10 μm. **d** The percentage of oocytes forming SINGs in control and salt stress treated worms as described in C. GFP::Ub control and GFP::UbAA control or salt stressed worms showed no signs of SING formation, whereas, GFP::Ub salt stressed did exhibit SING formation after stress exposure. A total of 510 oocytes were analyzed from 3 independent experiments (*n* = 30 worms). Statistical significance was calculated by a Fisher’s Exact test: ****p < 0.0001. **e** Young adult *C. elegans* expressing GFP::Ub and RPT-1::mCh were either soaked in M9 buffer or 500 mM NaCl for 60 min. Worms soaked in M9 did not form SINGs. Under salt stress conditions, worms without the proteasome inhibitor MG132 formed SINGs, whereas, in the presence of the proteasome inhibitor MG132, SING formation was reduced. Scale bar indicates 10 μm. **f** Proteasome inhibitors MG132, bortezomib, and lactacystin inhibit the formation of SINGs in response to salt stress. The percentage of oocytes forming SINGs after proteasome inhibitor treatment. A minimum of 240 oocytes were collected from 3 independent experiments (*n* = 24 worms). Statistical significance was calculated between salt stress and salt stress with proteasome inhibitor by a Fisher’s Exact test: ****p < 0.0001

Due to the presence of both ubiquitin and proteasome in the SINGs, we wanted to determine if the process of ubiquitination or proteasome activity was required for SING formation. First, we performed RNAi of the *uba-1* gene. This gene encodes the sole ubiquitin E1 enzyme in *C. elegans* [32]. RNAi of *uba-1*, reduced the formation of SINGs (Fig. 4a and b) suggesting that ubiquitination is required for SING formation.

To further explore ubiquitin chain formation at SING sites, live imaging on worms expressing an unconjugatable ubiquitin (GFP::UbAA) was performed (Fig. 4c and d). GFP::UbAA has a dialanine at the C-terminus rather than the diglycine that is required for conjugation onto protein substrates. SINGs were not present during stress in the GFP::UbAA strain indicating the ubiquitin conjugation was required for SING localization. This result along with *uba-1* result and the K48 antibody stain (Fig. 1d) indicate that SINGs contain ubiquitin that is conjugated onto substrates and that ubiquitination is required for ubiquitin and proteasome to localize to SINGs.

To evaluate whether proteasome activity is required for the formation of SINGs, proteasome inhibitors (MG132, Bortezomib, and Lactacystin) were used on worms expressing GFP::Ub and RPT-1::mCh. When worms were soaked in salt solution containing proteasome inhibitors, GFP::Ub and RPT-1::mCh fail to localize to SINGs (Fig. 4e and f). The result indicates that proteasome activity is required for ubiquitin and proteasome to localize to SINGs and was unexpected. Since ubiquitin localization to the SINGs precedes proteasomes (Additional file 4: Figure S3), it was anticipated that proteasomal activity would not be required for ubiquitin localization to SINGs. The proteasome inhibition results may indicate that there is a proteolytic event that is required in order to initiate the formation of the SINGs. Another likely explanation is that proteasome inhibition causes an overall decline in the amount of free ubiquitin and thus prevents any new ubiquitination events and SING formation.

Next, we explored the order of ubiquitin and proteasome appearance in the SINGs. To determine this, time-lapse studies of SING formation were conducted by collecting 30 consecutive frames with 1 min intervals on stressed *C. elegans* gonads from the strain expressing GFP::Ub and RPT-1::mCh. Each frame was assessed for the presence of ubiquitin, proteasome, or both in SINGs. SINGs with either ubiquitin only or both ubiquitin and proteasome were observed (Additional file 4: Figures S3). However, no SINGs were observed that contained only proteasome. Therefore, ubiquitin appears to precede the proteasome in SING formation.

**Fig. 5:**
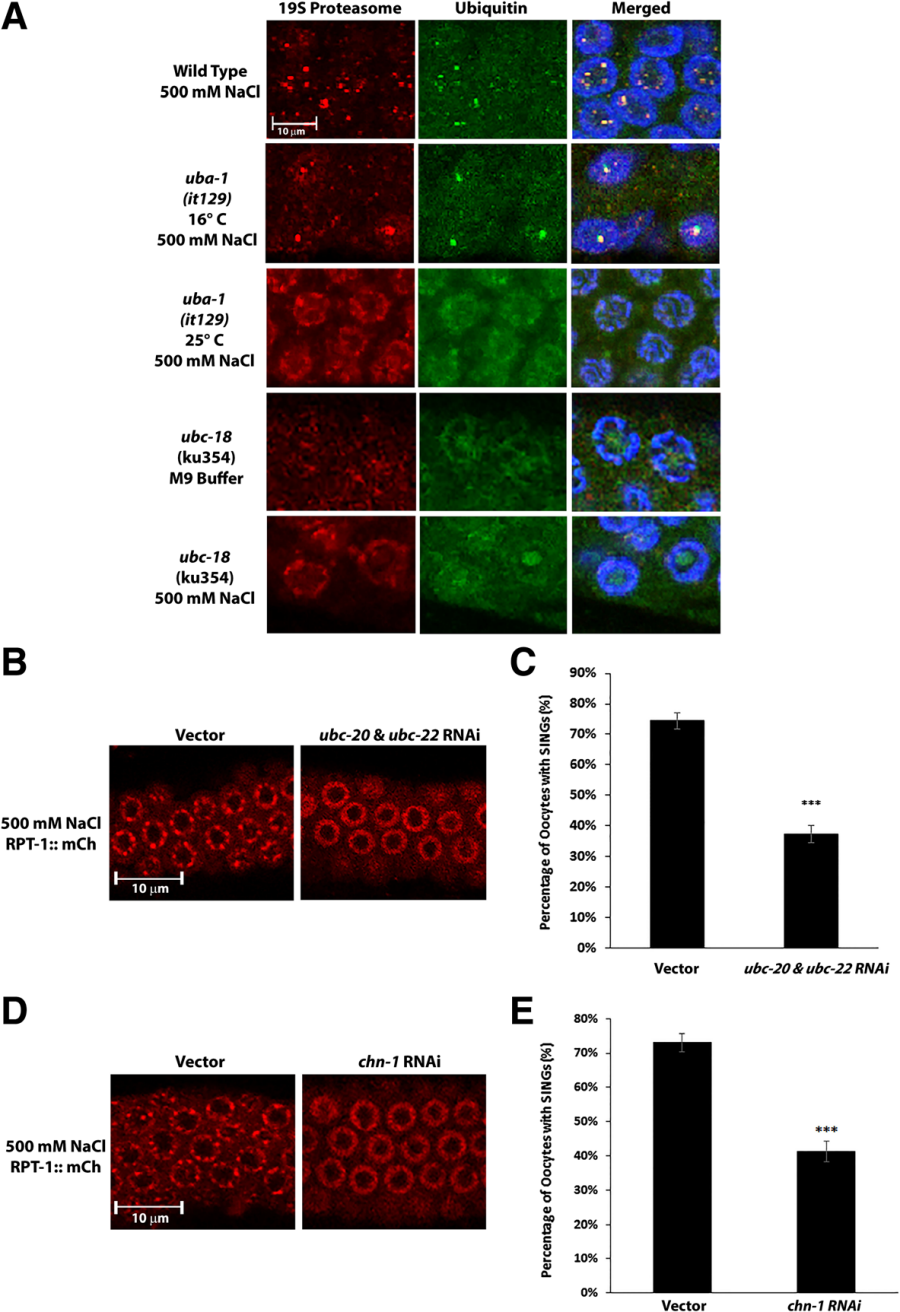
Ubiquitination pathway components participating in SING formation. **a** SINGs in *uba-1* and *ubc-18* mutants. Antibodies to ubiquitin and the 19S proteasome were used to stain wild type worms, a temperature sensitive mutant of *uba-1,* and a loss of function mutation in *ubc-18.* The merged images show the ubiquitin, proteasome, and DAPI channels. *uba-1* worms grown at 16 °C showed the presence of SINGs (551/600 oocytes) in response to salt stress (500 mM NaCl for 60 min). However, *uba-1* worms grown at 25 °C showed a reduction in SING formation (12/600 oocytes) when exposed to salt stress. SINGs were not induced in *ubc-18* mutants during salt stress (0/600 oocytes). Data were collected from 3 independent experiments (*n* = 30 worms). Scale bar indicates 10 μm. **b** Worms expressing RPT-1::mCh were subjected to either control RNAi (vector) or RNAi of the *ubc-20* plus *ubc-22* E2 enzymes. This combined RNAi reduced the appearance of SINGs. Scale bar indicates 10 μm. **c** Quantification of the percentage of oocytes forming SINGs after control RNAi and *chn-*1 RNAi treatment as described in B. A total of 1020 oocytes were observed from 3 independent experiments (*n* = 30 worms). Statistical significance was calculated by a two-tailed *z* test: ***p < 0.001. **d** Worms expressing RPT-1::mCh were subjected to either control RNAi (vector) or RNAi of the *chn-1* E3 enzyme. Knockdown of *chn-1* reduced the appearance of SINGs. Scale bar indicates 10 μm. **e** Quantification of the percentage of oocytes forming SINGs after treatment as described in D. A total of 1020 oocytes were observed from 3 independent experiments (*n* = 30 worms). Statistical significance was calculated by a two-tailed *z* test: ***p < 0.001

After establishing the requirement for ubiquitination and proteasome activity, we wanted to elucidate other aspects of the enzymatic pathway leading to SING formation. As confirmation that the E1 enzyme, *uba-1*, is required for SINGs formation we used a temperature sensitive mutation in the essential *uba-1* gene [32]. Under permissive conditions (16 °C) this mutant forms SINGs upon salt stress. Under nonpermissive conditions (25 °C), SINGs did not form after salt stress (Fig. 5a). In order to determine which E2 enzyme was required, an RNAi screen of the 24 ubiquitin E2 enzymes in *C. elegans* was conducted on stressed worms expressing GFP::Ub and mCh::H2B. RNAi of *ubc-18* inhibited the localization of GFP::Ub to SINGs (data not shown). Antibody staining for ubiquitin and 19S proteasome on *ubc-18(ku354)* mutant worms confirmed that *ubc-18* is required for SINGs formation after stress (Fig. 5a). UBC-18 is most similar to human UBE2L3 (a.k.a. UbcH7) which has been implicated in DNA repair and mitophagy [33, 34]. UBE2L3 is known to work specifically with HECT and RBR type E3 ubiquitin ligases [35] suggesting that this type of E3 might be involved in SING formation. In addition to the *ubc-18* result, we found that RNAi of a combination of both *ubc-20* and *ubc-22* caused a reduction in the number of oocytes with SINGs (Fig. 5b and c). UBC-20 and UBC-22 are both homologs of the human E2, UBE2K, which is known to be capable of K48 linked chain formation [36]*.*

Screens for E3s involved in the SING pathway revealed that knockdown of *chn-1* inhibited the formation of SINGs (Fig. 5d and e). *chn-1* is a homolog of CHIP which is a co-chaperone for Hsp70 and is known to be involved in the ubiquitination of misfolded proteins [37]. Thus, UBC-18, UBC-20/-22, and CHN-1 are all implicated in the ubiquitination of proteins that are required for or targeted to SINGs.

### SINGs are induced by a variety of stressors

**Fig. 6:**
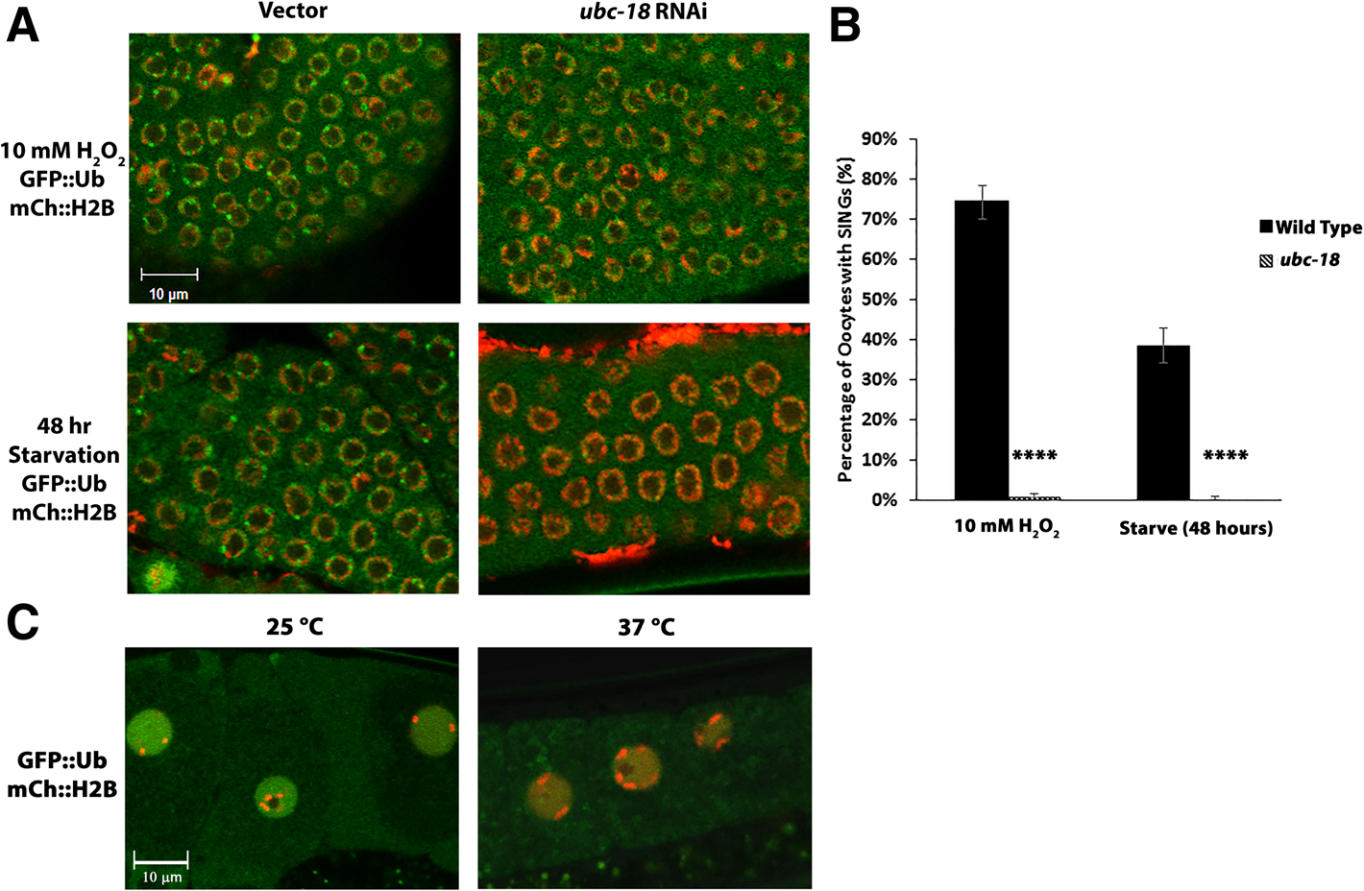
Oxidative stress and Starvation induce SING formation. **a** Oxidative stress and starvation in gonads. Worms expressing GFP::Ub and mCh::H2B were grown on bacteria with control or *ubc-18* RNAi and subjected to 10 mM H_2_O_2_ for 30 min or starvation for 48 h. Distal gonads are shown. Oxidative stress and starvation induced SING formation in vector treated worms. SINGs did not form in *ubc-18* worms soaked in H_2_O_2_ or starved. Scale bar indicates 10 μm. **b** Quantification of the percentage of oocytes forming SINGs after treatment as described in A. A total of 250 oocytes were collected from 3 independent experiments (*n* = 15 worms). Statistical significance was calculated by a two-tailed *z* test: ****p < 0.0001. **c** Heat shock does not induce SINGs. Worms expressing GFP::Ub and mCh::H2B were subjected to heat shock by placing them at 37 °C for one hour. In control worms incubated at 25 °C for 1 h, 0/600 oocytes showed SINGs. In the heat shock group, 0/600 oocytes showed SINGs. Data were collected from 2 independent experiments (*n* = 20 worms). Scale bar indicates 10 μm

In order to test if stressors other than high salt could induce SINGs, young adult worms expressing GFP::Ub and mCh::H2B were either soaked in 10 mM H_2_O_2_ for 60 min to induce oxidative stress, or grown under starvation conditions on NGM media without bacteria and peptone. Both H_2_O_2_ and starvation induced the formation of SINGs in oocytes (Fig. 6a and b). Thus, in addition to salt stress, both oxidative stress and starvation generate SINGs. These results show that SINGs are not a specific response to osmotic stress but seem to be involved in a general response to stressors that impair protein quality.

In order to test whether *ubc-18* was required for SING formation under these stress conditions, we treated worms with *ubc-18 (RNAi)* and exposed them to the same stress conditions. *ubc-18 (RNAi)* treated worms did not form SINGs under either stress condition (Fig. 6a and b). Therefore, *ubc-18* appears to be a general requirement for the formation of SINGs and is not specific to salt stress.

Heat shock is also a type of cellular stress. In order to examine whether heat shock induces the formation of SINGs, GFP::Ub and RPT-1::mCh worms were moved to 37 °C for 60 min, and then observed under confocal microscopy for the presence of SINGs. As a control, worms expressing HSF-1::GFP were subjected to the same procedure (data not shown). Consistent with a previous report [38] heat shock bodies were observed in the muscle nuclei of HSF-1::GFP worms (12/20 muscle nuclei showed heat shock bodies). However, in worms expressing GFP::Ub, no SINGs were present in the nuclei of the oocytes after heat shock (Fig. 6c). Therefore, these heat shock conditions do not induce SING formation.

Other studies have identified the transcription factor SKN-1 as an important component of the stress response in *C. elegans* [39, 40]. We investigated whether *skn-1* was required for the formation of SINGs. Antibody staining to detect SINGs was done on wild type and *skn-1(zu129)* mutant worms soaked in M9 buffer, 500 mM NaCl, or 10 mM H_2_O_2_. The nuclei of *skn-1* mutants formed SINGs in response to both types of stress (Additional file 5: Figure S4). Our results show that the SINGs seen during osmotic and oxidative stress are working through a stress response pathway that does not require *skn-1.*

**Fig. 7:**
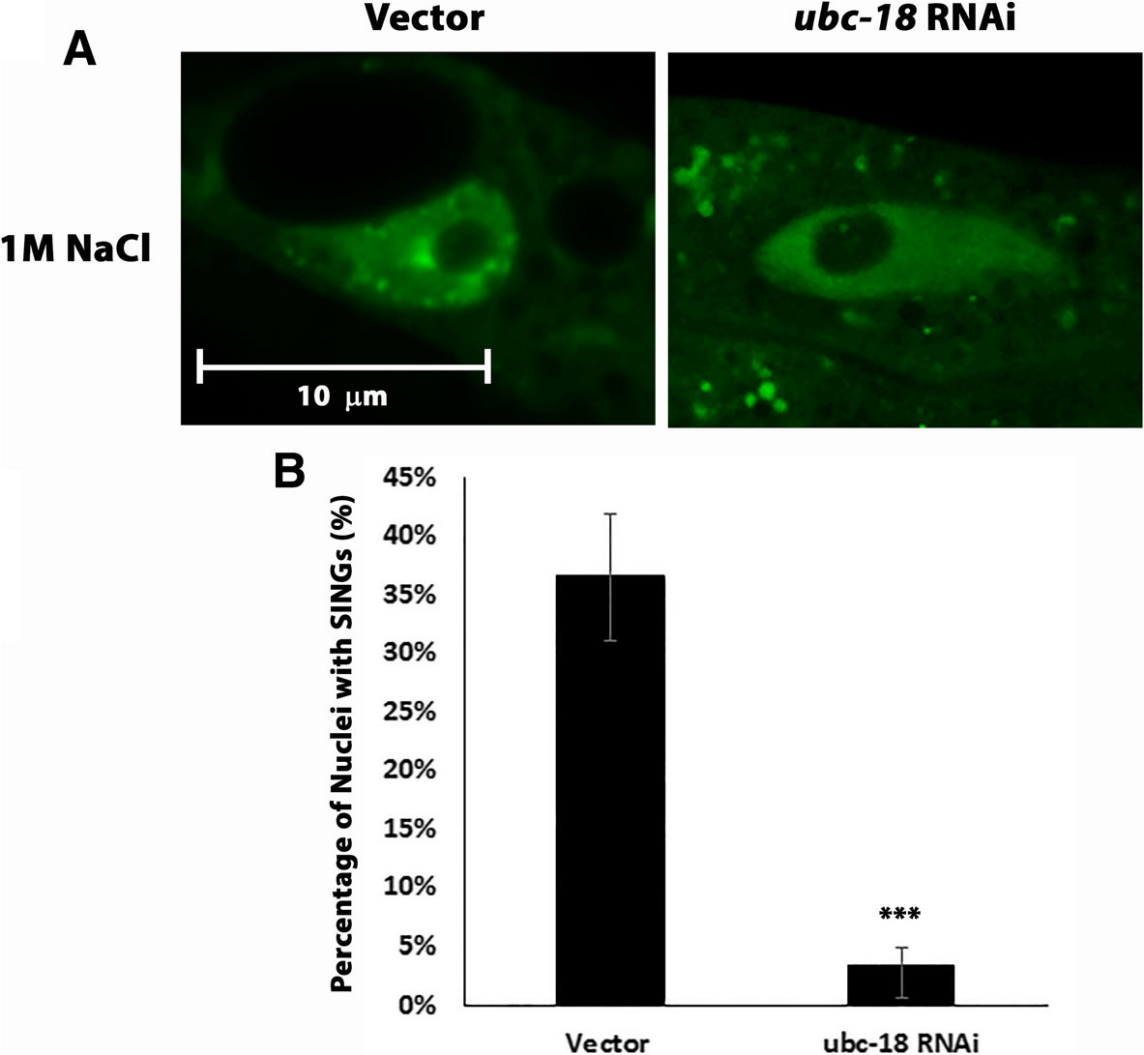
SINGs form in intestinal cells. **a** A strain expressing GFP::Ub in intestinal cells was soaked in 1 M NaCl for 1 h prior to imaging with a laser scanning confocal microscope. The salt stress induced SING formation in intestinal nuclei. Worms that had been grown under *ubc-18* (*RNAi*) did not form SINGs upon salt stress. **b** Quantification of the intestinal nuclear response to salt stress. Three trials were done for each condition including 10 worms per trial and 10 intestinal nuclei per worm (*n* = 30 worms). Statistical significance was calculated by a Fisher’s Exact test: ***p < 0.001

Nuclei of the oocytes differ from nuclei in other tissues in that they are engaged in the process of meiosis. DNA in oocyte nuclei is condensed as can be seen with the mCh::H2B reporter (Figs. 4c and 6c). Therefore, we wanted to investigate whether the SING phenomenon occurred in other tissues. Figure 7 shows intestinal nuclei expressing GFP::Ub. When worms are soaked in 1 M NaCl for one hour, intestinal cells form SINGs similar to what we have seen in oocytes. We have also seen SINGS form in muscle in response to salt and oxidative stress (Jacob Sanders and L.B, unpublished data). Therefore, this phenomenon is not restricted to the germline and may represent a general cellular response to stress.

**Fig. 8:**
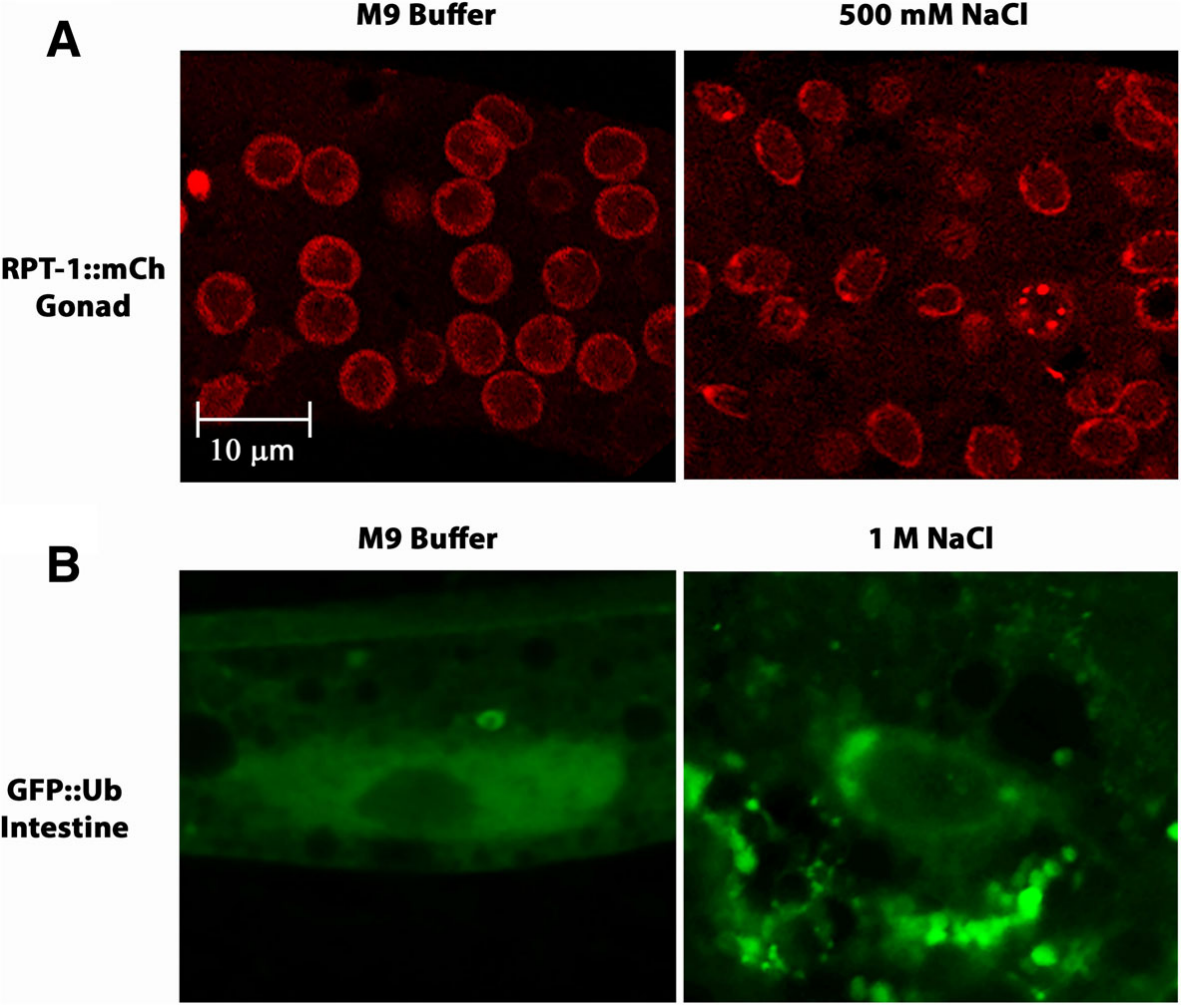
SING formation does not require intertissue signaling. **a** Young adult worms expressing RPT-1::mCh were cut open in either M9 buffer or 500 mM NaCl to extrude the gonads from the body. Carcasses containing the other tissues were quickly removed. Gonads were imaged after 60 min. All gonads soaked in 500 mM NaCl formed SINGs (134/300 nuclei; 10 gonads). No SINGs were formed in the gonads soaked in M9 (0/300 nuclei; 10 gonads). Data were collected from 3 independent experiments (*n* = 10 worms). **b** Intestinal cells also form SINGs when separated from the body. Intestinal nuclei are large and polyploid and have an elongated shape. Worms expressing GFP::Ub in the intestine were cut open in either M9 buffer or 1 M NaCl to extrude the intestine from the body. Carcasses containing the other tissues were quickly removed. Intestines were imaged after 60 min. No intestines soaked in M9 formed SINGs (0/40 nuclei; 8 intestines). For intestines soaked in 1 M NaCl, all intestines showed SINGs (14/30 nuclei; 8 intestines). Data were collected from 2 independent experiments (*n* = 8 worms). Scale bar indicates 10 μm

Several recent studies have shown that some stress responses in *C. elegans* involve intertissue signaling (reviewed in [41]). In order to test the possibility that SINGs may be forming in response to a systemic signaling mechanism, worms were cut open in salt solution and the intestine or the gonad was liberated from the body of the worm. The carcasses were quickly removed to eliminate any signaling from other tissues. Under these conditions, SING formation proceeded normally in both the gonad and the intestine (Fig. 8). Therefore, the SING response is tissue autonomous and does not require signaling from other tissues.

### TIAR-2 is a component of SINGs

**Fig. 9:**
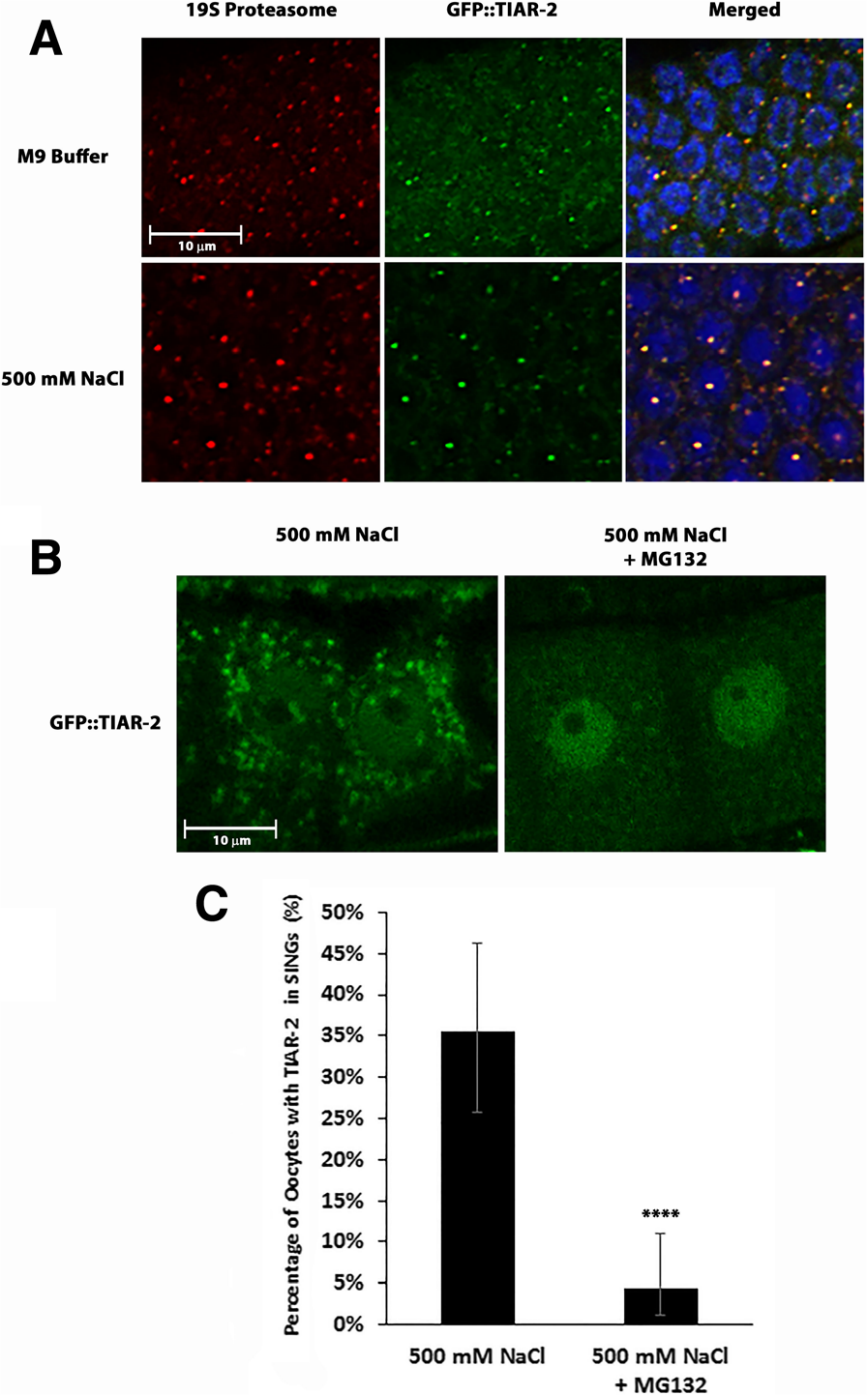
SINGs contain the TIAR-2 protein. **a** Worms expressing GFP::TIAR-2 were soaked in M9 or 500 mM NaCl for 60 min and then stained with an antibody to the 19S proteasome. The merged image shows the TIAR-2, proteasome, and DAPI channels. In M9 treated worms, few oocytes showed nuclei with TIAR-2 and proteasome colocalization (38/600). Whereas, in salt stressed worms, the number of TIAR-2 and proteasome colocalization events increased (212/600 oocytes). Data were collected from 3 independent experiments (*n* = 30 worms). Scale bar indicates 10 μm. **b** Day 4 adults expressing TIAR-2::GFP were soaked in a watch glass containing 500 mM NaCl with or without MG132 for 60 min. Worms that were not treated with MG132 showed TIAR-2 in SINGs, whereas MG132 treated worms showed fewer TIAR-2 containing SINGs. **c** Quantification of the percentage of TIAR-2 in SINGs as described in B. A total of 90 proximal oocytes were observed from 3 independent experiments (*n* = 30 worms). Statistical significance was calculated by a Fisher’s Exact test: ****p < 0.0001

In 2008, Jud et al published that the RNA binding protein TIAR-2 (a.k.a. TIA-1) localized to nuclear granules in response to salt stress [42]. TIAR-2 is one of three proteins in *C. elegans* that are similar to the TIA1/TIAR proteins from mammals. These proteins are involved in splicing and they also interact with untranslated mRNAs during stress conditions [43, 44]. TIA1/TIAR proteins localize to cytoplasmic stress granules in mammalian cells. Experiments in *C. elegans* have shown that two of the homologs, TIAR-1 and TIAR-2 also localize to cytoplasmic granules in response to proteotoxic stress [42, 45]. TIAR-2 is more highly enriched in the nucleus in both stressed and unstressed conditions. We found that a TIAR-2::GFP reporter colocalizes with endogenous proteasome in SINGs after salt stress (Fig. 9a). Like ubiquitin and the proteasome itself, TIAR-2 presence at SINGs was dependent upon proteasome activity (Fig. 9b and c).

Other proteins were also tested for their presence in SINGs. Live imaging was conducted on fluorescently labeled nuclear pore proteins (NPPs), tubulin, SUMO, and the PGL-1 protein in the germline of *C. elegans*. Results showed that some NPPs did react to salt stress by forming perinuclear foci, but these areas did not colocalize with SINGs (Additional file 3: Figure S5A and S5B). Tubulin remained in the cytoplasm and exhibited minor rearrangement during salt stress (Additional file 3: Figure S5D). SUMO (SMO-1) and PGL-1, a P granule component also do not localize to SINGs after salt stress (Additional file 3: Figure S5C and S5D). Therefore, relocalization to SINGs is not a general phenomenon for all proteins in the oocyte. Also, SINGs do not contain high concentration of RNA based on staining with SYTO 14 after stress induction (Additional file 3: Figure S5E).

### Embryos containing SINGs exhibit embryonic lethality and impaired cell division

**Fig. 10:**
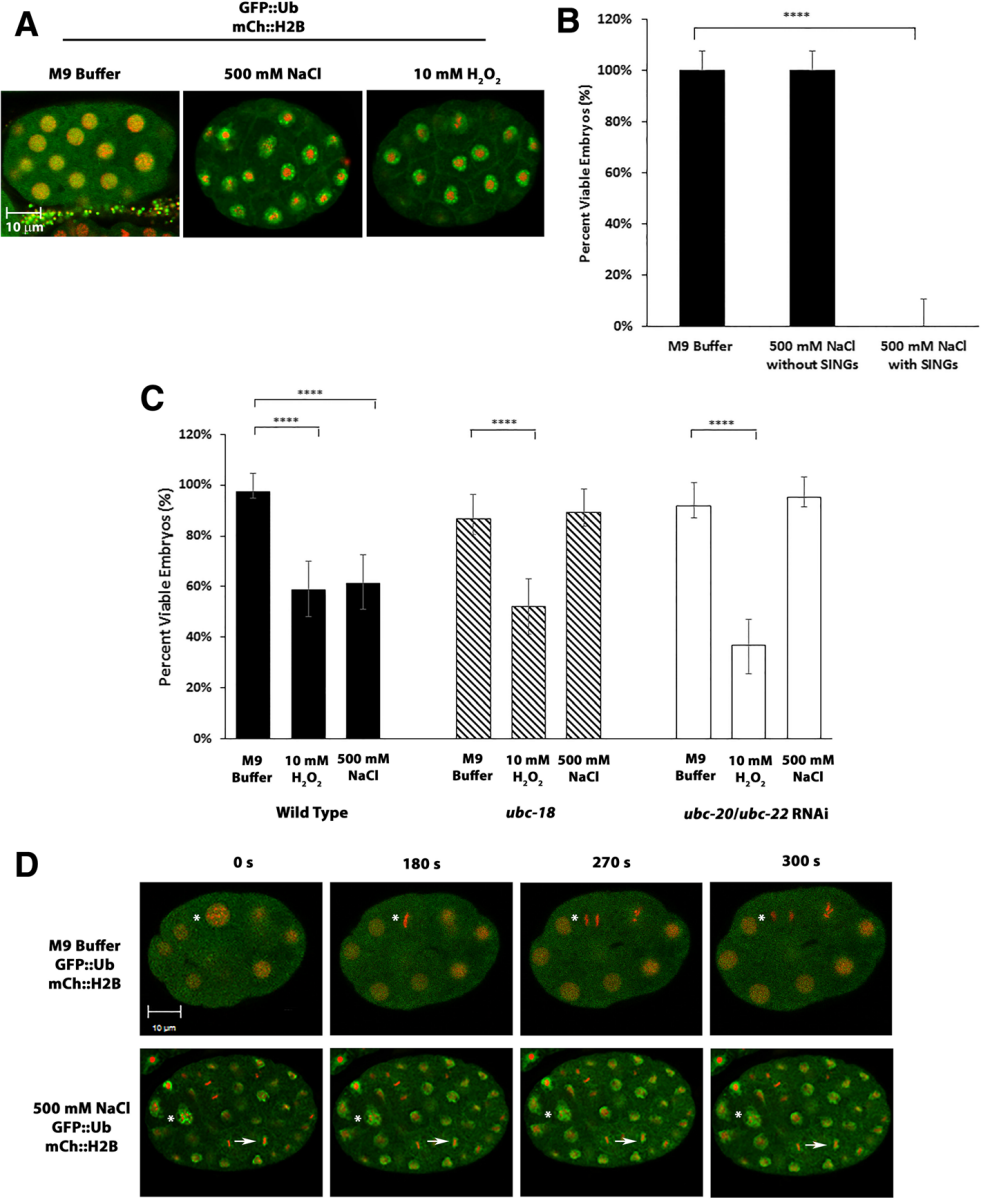
SINGs correlate with embryonic lethality and impaired cell division. **a** Stress induces SING formation in embryos. Embryos were obtained from worms expressing GFP::Ub and mCh::H2B and placed in 500 mM NaCl for 1 h or 10 mM H_2_0_2_ for 30 min. Embryos formed SINGs under both types of stress conditions. Scale bar indicates 10 μm. **b** Hatching rate of embryos that contain SINGs. Embryos were dissected from gravid adults that were soaked in M9 (unstressed) or 500 mM NaCl (stressed) for 1 h. Stressed embryos were screened under the microscope for the presence of SINGs. Embryos with SINGs showed a 0% hatch rate versus a 100% hatch rate for unstressed embryos or stressed embryos without SINGs. A total of 40 embryos were analyzed for each condition from 3 independent experiments. Statistical significance was calculated by a Fisher’s Exact test: ****p < 0.0001. **c** Hatching rates of wild type and *ubc-18* mutant embryos in unstressed and stressed conditions. Embryos were subjected to two different stresses: salt stress (500 mM NaCl for 60 min) and oxidative stress (10 mM H_2_O_2_ for 30 min). Both types of stress reduce the hatching rate of wild type embryos. However, in *ubc-18* or *ubc-20/ubc-22 (RNAi)* embryos which have reduced SING formation, salt stress does not reduce the hatching rate. Oxidative stress does reduce hatching rate in *ubc-18* and *ubc-20/ubc-22 (RNAi)* embryos. In *ubc-18* and *ubc-20/ubc-22 (RNAi)* embryos, the numbers for the M9 and the 500 mM NaCl hatching rates showed no statistical difference. Experiments were repeated three times with 25 embryos per trial for a total of 75 embryos per condition. Statistical significance was calculated by a Fisher’s Exact test: ****p < 0.0001. **d** Time-lapse imaging on unstressed and stressed embryos expressing GFP::Ub and mCh::H2B. Ten embryos from the unstressed and ten embryos from the salt stressed groups were observed on the confocal microscope with time-lapse imaging for 15 min each. Many cells in each unstressed embryo were able to complete cell division within 5 min. Cells in salt stressed embryos showed no progression through the cell division cycle. The asterisk in the M9 embryo shows a cell that proceeds through prophase, metaphase, and anaphase of the cell cycle. The asterisk in the 500 mM NaCl embryo shows a nucleus that has SINGs and remains in prophase. The arrow shows a nucleus with SINGs that remains in metaphase throughout the 15 min observation period. A total of 10 embryos were collected from 3 independent experiments. Scale bar indicates 10 μm

SINGs are induced in embryos that are exposed to salt or oxidative stress (Fig. 10a). Therefore, we wanted to determine if there was a correlation between SING formation and embryonic lethality. Embryos from worms expressing GFP::Ub and RPT-1::mCh were salt stressed and then selected for the presence or absence of SINGs before placing them on a seeded plate to monitor hatching after 48 h. 100% of the embryos that were unstressed hatched as did stressed embryos that were devoid of SINGs; whereas, 0% of embryos that were stressed and contained SINGs hatched (Fig. 10b). Thus, there is a correlation between the presence of SINGs and embryonic lethality.

Since *ubc-18* loss of function reduces the formation of SINGs, we went on to test if *ubc-18* embryos showed any resistance to stress conditions. Wild type and *ubc-18* mutant embryos were placed under unstressed or salt stress conditions, and then scored for hatching after 48 h. 97% of the wild type unstressed embryos hatched and 61% of the wild type salt stressed embryos hatched (Fig. 10c). This result was expected because approximately half of stressed embryos exhibit SING formation. In unstressed *ubc-18* mutants 86% of the embryos hatched; however, 89% of the stressed *ubc-18* mutant embryos were able to hatch (Fig. 10c). These data suggest that *ubc-18* embryos have a lower hatching rate than wild type, but that they are more resistant to salt stress. Prior studies with *ubc-18* had shown a reduced brood size, but the actual hatching rate had not been reported [46]. No association between *ubc-18* and an elevated resistance to stress has been reported previously.

Similarly to *ubc-18,* RNAi knockdown of *ubc-20* plus *ubc-22* reduces SING formation (Fig. 5b and c). We found that RNAi of *ubc-20/ubc-22* also increased embryonic resistance to salt stress (Fig. 10c). The correlation between the lack of SINGs and the increased embryonic survival in mutant or RNAi embryos supports the hypothesis that SINGs have a negative impact on cell survival or development. Interestingly, *ubc-18* and *ubc-20/22* embryos do not survive better than wild type under oxidative stress (Fig. 10c). This could be because oxidative stress has additional detrimental effects other than the induction of SINGs.

In order to understand the nature of the embryonic lethality that is seen with salt stress, time-lapse imaging was performed on embryos expressing GFP::Ub and mCh::H2B. Embryos were soaked in either M9 (unstressed) or 500 mM NaCl (stressed) prior to imaging for 15 min. The cells of unstressed embryos were able to undergo a complete cell division cycle within 15 min (Fig. 10d; Additional file 6: Movie S1). However, the cells of stressed embryos did not complete cell division and appeared to be halted at a variety of stages in the cell cycle (Fig. 10d; Additional file 7: Movie S2). Out of the 10 salt stressed embryos that were observed, none showed any cells that progressed through any cell cycle phases. Thus, the embryonic lethality that is seen in embryos after salt stress is likely due to interference with the cell cycle. Since stressed embryos that do not contain SINGs are able to undergo development (Fig. 10b), this suggests that the SINGs themselves may have a negative influence on cell division.Additional file 6:
**Movie S1.** GFP::Ub and mCh::H2B embryos soaked in M9 buffer for 60 min. Unstressed embryos undergo complete cell division within 5 min. (AVI 2889 kb)
Additional file 7:
**Movie S2.** GFP::Ub and mCh::H2B embryos soaked in 500 mM NaCl for 60 min. Salt stressed embryos do not complete cell division. (AVI 4369 kb)


Exposure to stress is also known to affect the lifespan of an organism [47]. The oocyte SINGs persist for up to 72 h post stress exposure (data not shown) and thus it is presumed that they may have long term effects on the organism. We tested whether salt stress had an effect on the lifespan of *C. elegans*. Life span studies were carried out on adult wild type and *ubc-18* (*RNAi*) worms that had been exposed to 500 mM NaCl for 60 min as L4 larvae. Surprisingly, this treatment had no observable effect on the lifespan (Additional file 8: Figure S6).

## Discussion

Our results reveal a novel pathway that responds to collapse of protein quality in the germline and other tissues of *C. elegans.* Salt stress, oxidative stress, and starvation lead to the redistribution of ubiquitin, proteasome, and the TIAR-2 protein into structures we refer to as Stress Induced Nuclear Granules.

Environmental stress has previously been shown to induce various nuclear bodies. One example are the heat shock bodies containing HSF1. Insulator bodies form in *Drosophila* cells in response to osmotic stress however they localize to chromatin [16]. The *C. elegans* SINGs are similar to clastosomes and PML bodies in that they contain ubiquitin and proteasome and are induced by stress [15, 19, 20, 48]. However, PML bodies have a high concentration of SUMO which is not present in SINGs. Clastosomes which were reported to form in mammalian cultured cells appear to be the most similar to the SINGs since they also require proteasome activity to form [15].

**Fig. 11:**
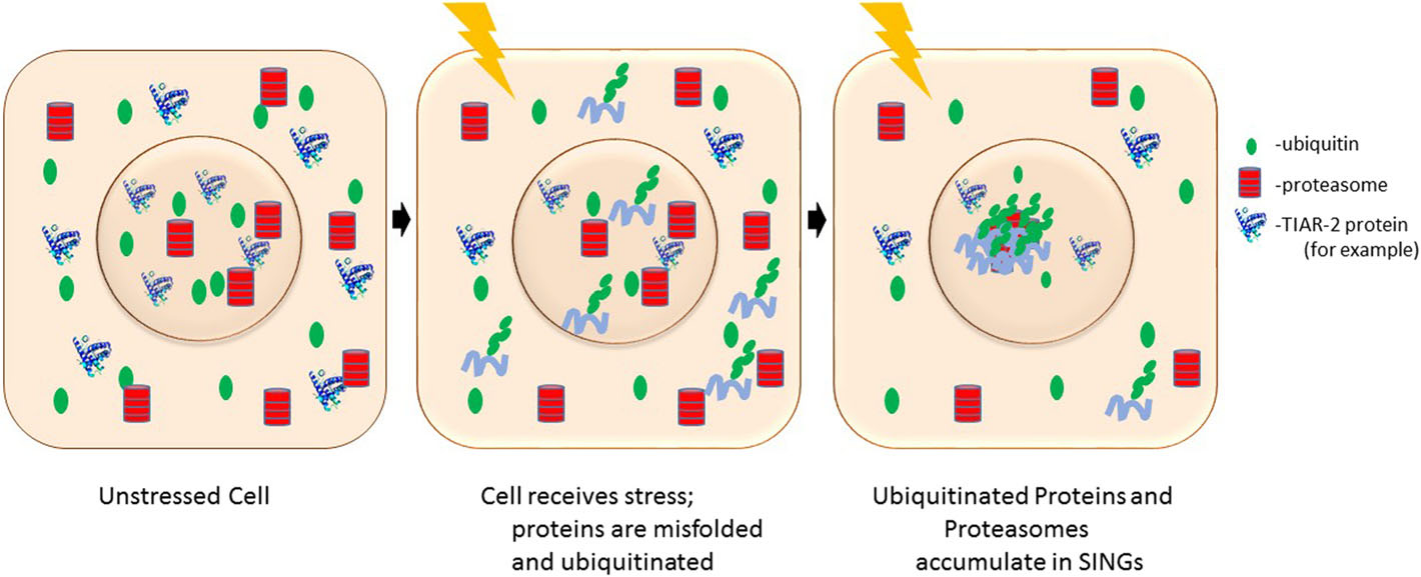
Model for SING formation. Unstressed cells experience a healthy protein homeostasis. When cells are exposed to stressors that induce protein misfolding, the ubiquitin system adds K48-linked ubiquitin chains to the misfolded proteins. Ubiquitinated proteins and proteasomes localize to SINGs in the nucleus

The finding that a brief heat shock can abrogate the formation of SINGs suggested that induction of chaperones might prevent SINGs formation. The requirement for *hsf-1* in this phenomenon further supports that model. Altogether our results suggest a model where stress causes protein damage or misfolding which triggers ubiquitination of damaged proteins and the accumulation of those proteins along with proteasomes at nuclear foci (Fig. 11).

An interesting aspect of our findings is that proteasome and ubiquitin foci form inside the nucleus rather than in the cytoplasm. This result adds to a growing number of reports that suggest that protein degradation is taking place in the nucleus (reviewed in [49]). In yeast, it has been reported that degradation of some short-lived proteins occurs by first importing them into the nucleus where they are degraded by nuclear proteasomes [10]. The presence of the proteasome and K48-linked ubiquitin chains is consistent with the idea that SINGs are sites of localized protein degradation. If protein degradation is occurring at the SINGs, it will be of great interest to determine whether there is a specific set of proteins that are degraded or whether SINGs are general sites of degradation for any K48 ubiquitinated protein. Follow up studies are underway in an attempt to discriminate between these two possibilities.

SING formation in embryos results in a cell cycle arrest. Thus, SINGs may interfere with cell division. Embryos that lack *ubc-18* do not exhibit SINGs when stressed and they can proceed through the cell cycle normally. This may point to a direct role of the SINGs in compromising cell division. Further studies are needed in order to determine which aspects of cell division are affected when SINGs are present. This effect of stress on the cell cycle during embryogenesis may be a contributing factor to compromised reproductive capability in organisms exposed to environmental stress [50].

## Conclusions

Our studies have identified a novel nuclear structure that we refer to as SINGs. Salt stress, osmotic stress, and starvation induce SING formation. SINGs share some features with previously identified nuclear bodies such as clastosomes and PML bodies. The appearance of SINGs correlates with conditions of protein misfolding. Thus, SINGs likely participate in a nuclear protein quality control pathway that is initiated when misfolded proteins accumulate. Ubiquitination and nuclear import are required for the formation of SINGs suggesting that misfolded proteins from the cytoplasm may be transported into the nucleus before localizing to SINGs. Future research will explore whether ubiquitination occurs prior to nuclear import or after.

## Methods

### C. elegans *Strains and Maintenance*

*C. elegans* strains were cultured on nematode growth medium (NGM) with a bacterial lawn of *E. coli* strain OP50 and incubated at 20 °C, 25 °C, or 16 °C. Wild type and *ubc-18* mutants were grown at 20 °C and transgenic worms were incubated at 25 °C. Temperature sensitive mutant strain (RV110) was grown that 16 °C and shifted to 25 °C for 18 h before stress was administered. Strains used in this study are listed in Table 1.

**Table 1:** *C. elegans* strains used in the study

Strain name	Allele(s)
AZ244	*ruls57* [*pie-1*::GFP::tubulin + *unc-119*(+)]
BC10060	*sEx884* [rCes*C12C8.1*::GFP + pCeh361]
ERT261	*jyEx128* [*vha*-*6p*::GFP::Ub cb-*unc-119*(+)]; ttTi5605 II; *unc*-*119*(ed9)
EU40	*skn-1*(*zu129*) IV/nT1 [*unc*-?(*n754*) *let*-?] (IV; V).
FGP8	*fgpIs20* [(pFGP79) *pie-1p*::mCherry::*smo-1*(GG) + *unc-119*(+)]; *ruIs32* [*pie-1p*::GFP::H2B + *unc-119(*+)] III
JH1327	*axEx73* [*pie-1*p::*pie-1*::GFP + *rol-6(su1006)* + N2 genomic DNA]
JH2099	*axIs1486* [pCG51; LAP::*Y46G5A.13*(*tia-1.2*) +*unc-119*(+)]
JH2184	*axls1595*[*pie-1p*::GFP::*npp-*9(orf)::*npp-9* 3'UTR + *unc-119*(+)]
JH2458	*axIs1735*[*pie-1p*::LAP tag::*npp-10* (full length) + *unc-119* (+)]
JH2842	*ltIs37* [(pAA64) pie-1p::mCherry::*his-58* + *unc-119*(+)] IV; *axIs1522* [pie-1p::GFP::*pgl-1*::*pgl-1* 3'UTR + *unc-119*(+)]
JH2686	*axIs1844*[GFP:: *npp-7* + *unc-119*(+)]
LN130	*rcIs31* [*pie-1p::*GFP::Ub *+ unc-119*(+)]*; ltIs37* [*pie-1p*::mCherry::*his-58*]
LN154	*rcIs31* [*pie-1p::*GFP::Ub *+ unc-119*(+)]*; rcSi1 [mex-5p:*:*rpt-1*::mCherry *+ unc-119] II; unc-119 (ed3)*
LN162	*avIs116* [*pie-1p*∷GFP∷UbAA *+ unc-119(+)*]; *ltIs37* [*pie-1p*::mCherry::*his-58*]
LW1089	*jjls1089* [*npp-1*::GFP + *unc-119*(+)]
N2 (Bristol)	Wild Type
OG497	*drSi13 [hsf-1p::hsf-1*::GFP::*unc-54*' 3'UTR + *Cbr-unc-119*(+)] II
RV110	*uba-1(it129) IV*
WY34	*ubc-18(ku354) III*
XA3546	*qaIs3546* [*unc-119*(+) + *pie-1*::GFP::*npp-8*]; *unc-119*(ed3)

### Fluorescence microscopy and time-lapse imaging

All fluorescent and time-lapse images were acquired using a ZEISS AxioObserver with a LSM 700 confocal module and a 63X/1.4 Plan-Apochromat oil DIC M27 objective. Images were analyzed with ZEN 2009 software. In live imaging and antibody staining experiments, the 488 nm laser was used for imaging both GFP and FITC, and the 555 nm laser was used for both mCherry and TRITC. DAPI was imaged using the 405 nm laser. Image settings on the microscope were kept constant for each set of experiments. Dimensions of images acquired were 512 × 512 pixels at a depth of 8 bits. Images were adjusted to be brighter for visual presentation in Photoshop using the brightness function.

A time-lapse series (30 frames with 1 min intervals) of the worm strain LN154 (GFP::Ub and RPT-1:: mCh) was used to investigate the timing of colocalization events. All time-lapse experiments were imaged on the confocal at room temperature. A time-lapse series (30 frames with 0.5 min intervals) of LN130 (GFP::Ub and mCh::H2B) was used for cell division experiments.

### Antibodies

Primary antibodies used were rabbit anti-19S proteasome (sc-98797 from Santa Cruz), mouse monoclonal anti-20S proteasome (MCP20 from Enzo Lifesciences), mouse monoclonal anti-ubiquitin (P4D1 from Santa Cruz), rabbit anti-K48 ubiquitin (Apu2 from Millipore), and rabbit anti-K63 ubiquitin (Apu3 from Millipore). Secondary antibodies used were goat anti-mouse FITC (Abcam) and goat anti-rabbit TRITC (Jackson ImmunoResearch Labratories).

### *Immunostaining of* C. elegans

Day 1 adult worms were placed in Egg Buffer and cut open on poly-L-lysine-coated slides to release the gonadal arms and embryos. Slides were placed in liquid nitrogen and fixed with 100% methanol at -20 °C for 20 min, followed by washing three times with PBST for 5 min. Slides were then blocked for 1 h with 30% normal goat serum in PBST at 23 °C, and incubated overnight with primary antibody (1:200) at 4 °C. Slides were washed with PBST, and incubated with secondary antibody (1:200) at 23 °C for 1.5 h. Vectashield plus DAPI (Vector Labs, Burlingame, CA) was used for mounting each slide prior to imaging with confocal microcopy.

### FRAP Analysis

Photobleaching experiments were performed using a Zeiss LSM700 laser scanning confocal system. Two pre-bleach images were acquired. Bleaching was done via 40 iterations of bleaching with 100% on the 488 nm laser. Fluorescence was bleached to ≤ 20% of initial intensity. Images were acquired every 1 s with the 488 nm laser set to 5% power. Each data point was normalized for background and photobleaching using the equation: [(ROI_b_ – ROI_bg_)/(ROI_nb_ –ROI_bg_)]/[(pbROI_b_ –pbROI_bg_)/(pbROI_nb_ –pbROI_bg_)].

### RNAi by feeding

RNAi was achieved by feeding worms bacteria that express dsRNA for each gene. RNAi clones for the UBC genes were obtained from the Ahringer library or the Vidal ORF library. The *ubc-18* RNAi is an ORF clone. Controls for the RNAi experiments used the L4440 plasmid (vector) without any gene insert in the HT115 bacterial strain. RNAi clones were streaked from glycerol stocks onto tryptic soy agar media with ampicillin (100 μg/mL) and tetracycline (10 μg/mL). Bacterial overnights were grown in tryptic soy broth with ampicillin and tetracycline. NGM plates containing ampicillin and 0.2% lactose were seeded with the bacterial overnights. L4s were transferred to above plates and grown for 22 h under ideal conditions for each respective strain.

### Osmotic stress, oxidative stress, and starvation

*C. elegans* were grown on OP50 containing NGM at their appropriate temperatures until they reached 1 day adults. Worms from this population were then moved to control and stressed conditions. Conditions involving liquid (control, salt, osmotic, and oxidative stress) were performed by soaking day 1 adults in a watch glass containing 1 mL of solution at room temperature. Solution concentrations for each stress are as follows: 500 mM NaCl (salt stress), 815 mM Sucrose (osmotic stress), 10 mM H_2_O_2_ (oxidative stress). Control (unstressed) individuals were soaked M9 buffer for 60 min. The worm strain JH2099 (GFP:: TIAR-2) was salt stressed for 120 min. The GFP::Ub intestinal strain (ERT261) was stressed at 1 M NaCl for 60 min prior to imaging. Oxidative stress was induced by soaking worms in a 10 mM H_2_O_2_ solution for 60 min.

For starvation, synchronized L4 worms were moved to NGM plates without OP50 bacteria or peptone for a period of 48 h prior to being examined. To induce heat shock, day 1 adults grown on seeded NGM at 25 °C were shifted to a 37 °C for 60 min prior to imaging.

After treatment, worms were mounted on a 3% agar pad and examined with a laser scanning confocal microscope (Zeiss LSM700).

### Treatment with proteasome inhibitors

Proteasome inhibitor solutions were prepared the same day as the experiment by adding proteasome inhibitor stock solution to either M9 buffer or 500 mM NaCl. Proteasome inhibitors and concentrations tested include 20 μM MG132, 10 μM Lactacystin, and 20 μM Bortezomib. *In vivo* proteasome inhibitor experiments were performed by soaking day 1 adult worms in 20 μM MG132 for 60 min at room temperature. Worms were then transferred over to a solution containing both MG132 and 500 mM NaCl. Control worms were soaked in a 500 mM NaCl solution with no proteasome inhibitor for 60 min before imaging.

### *Detection of DNA and RNA in* C. elegans

The cell permeable SYTO14 green fluorescent nucleic acid stain (Life Technologies) was used to observe total RNA in oocytes. SYTO14-stained RNA excites at 521 nm and emits at 547 nm. Fresh SYTO14 solution was either made in M9 buffer (unstressed) or 500 mM NaCl (stressed). Worms were dissected in 5 μm SYTO14 solution for 15 min and then imaged by confocal microscopy.

DAPI (NucBlue Fixed Cell ReadyProbes, Life Technologies) was used in the SYTO14 experiment to visualize DNA. One drop of NucBlue was added directly to the top of the slide and incubated for 15 min prior to visualization.

### Prior heat shock exposure

Day 1 adult worms grown at 25 °C were shifted to 34 °C for 60 min prior to 500 mM NaCl exposure and then imaged with confocal microscopy. Control individuals stayed at the initial growing temperature before being exposed to salt stress.

### Embryonic lethality and cell division analysis

Wild type and *ubc-18* (*RNAi*) embryos were cut out of day 1 adult worms and soaked in either M9 buffer (control), 500 mM NaCl, or 10 mM H_2_O_2_ for 60 min. After which, embryos were then pipetted onto unseeded NGM plates and scored for hatching at 48 h.

Cell division analysis was conducted by taking time-lapse movies on both control and salt stressed embryos expressing GFP::Ub and mCh::H2B. Time-lapse movies were described above in the *Fluorescence Microscopy and Time-lapse Imaging* section.

### Longevity assays

L1 worms were moved to either vector or RNAi seeded plates. Once worms reached the L4 stage they were subjected to 500 mM NaCl stress for 60 min and then moved to new RNAi seeded NGM plates. 80 worms were used for each condition.

### Statistical analysis

Sample sizes and number of experiments performed are noted in each figure legend. In general, a minimum of 1020 oocytes were observed over three biological repeats unless otherwise noted in the figure legend. Two sample *z*-tests were performed using VasarStats on the 20S and TIAR-2 antibody stains as well as on live-cell images which include RNAi and stress experiments (Figs. 1e, 2b, 3b, d, 4b and 5c, e). The Fisher’s exact test was performed on data that was less than five (Figs. 2c, 4d, f, 6b and 10b, c, and Additional file 5: Figure S4B). Error bars presented in this paper represent a 95% confidence interval and were derived using the modified Wald method on GraphPad. Data was considered to be statistically significant if *p* < 0.05. A log-rank test was used to determine significant differences in survivorship curves in the lifespan experiment.

## Additional files

Additional file 1: Figure S1. High sucrose also induces SINGs. Young adult worms expressing GFP::Ub were soaked in either M9, 500 mM NaCl, or 815 mM sucrose for 1 h. Worms were then imaged on the confocal microscope and assessed for the presence of SINGs in oocyte nuclei. A higher concentration of sucrose was used due to the lower osmolarity of sucrose as compared to NaCl. For each condition, a total of 900 oocytes were observed from 3 independent experiments (*n* = 30 worms). Statistical significance was calculated by a Fisher’s Exact test: ***p < 0.001. (JPG 234 kb)

Additional file 2: Figure S2. FRAP analysis of SINGs. GFP::Ub in SINGs of the nuclei of salt stressed oocytes (blue) is compared to GFP::Ub in unstressed nuclei (green) and Q82::GFP in aggregates in muscle cells (red). The Q82::GFP shows little recovery of fluorescence over a one minute period, whereas, GFP::Ub in SINGs recovers to approximately 50% of initial level after 1 min. Graph shows the data from 10 individual FRAP experiments with standard errors indicated. (JPG 559 kb)

Additional file 3: Figure S5. Nuclear pore proteins, tubulin, PGL-1, and SMO-1 do not localize to SINGs during salt stress. (A) Worm strains expressing GFP::NPP were exposed to both M9 buffer and 500 mM NaCl for 60 min. The nuclear pore proteins responded to stress by occasionally forming concentrated areas of protein at the periphery of the nucleus: NPP-1 (19/250 oocytes) and NPP-7 (14/250 oocytes). A total of 250 oocytes were observed from 10 worms. (B) NPP-1 was crossed into a RPT-1::mCh expressing worm and then subjected to unstressed and salt stressed conditions. SINGs were not found to colocalize with NPP-1 (48/50 oocytes). A total of 50 proximal oocytes were observed from 10 worms. (C) Worms expressing GFP:: H2B and mCh:: *smo-1* were soaked in M9 and 500 mM NaCl for 60 min. SMO-1 localized to the nucleolus in control and salt stress groups, but did not localize to SINGs (80/80 oocytes). A total of 80 proximal oocytes were observed from 2 independent experiments (*n* = 20 worms). (D) Tubulin and PGL-1 do not localize to SINGs during salt stress. GFP::tubulin worms were soaked in M9 buffer or 500 mM NaCl for 60 min and then observed under confocal microcopy. Both unstressed and stressed GFP::tubulin did not localize to SINGs (50/50 oocytes for each condition). A total of 50 oocytes were collected from 10 worms. Minor rearrangement of cytoplasmic tubulin was seen in stressed GFP::tubulin populations. GFP:: PGL-1 worms were soaked in M9 buffer or 500 mM NaCl for 60 min and then observed under confocal microcopy. Both unstressed and stressed GFP::PGL-1 did not localize to SINGs (240/240 oocytes for each condition). A total of 240 oocytes were collected from 6 worms. (E) Worms were stained with the RNA dye, SYTO 14. In unstressed worms high concentrations of RNA are detected in the cytoplasm and in the nucleolus (80/80 oocytes). In stressed worms, RNA localizes to cytoplasmic stress granules, but not to SINGs in the nucleus (80/80 oocytes). A total of 80 oocytes were collected from two independent experiments (*n =* 10 worms). Scale bars indicate 10 μm. (JPG 1898 kb)

Additional file 4: Figure S3. Time-lapse analysis of SINGs. Worms expressing GFP::Ub (green) and RPT-1::mCh (red) and soaked in 500 mM NaCl for 60 min were imaged for 30 min with an image taken every minute. When SINGs first appeared in the time-lapse series, they were assessed for the presence ubiquitin, RPT-1, or both ubiquitin and RPT-1. The numbers for each category are shown in the graph. RPT-1 alone was not observed at any of the initial SING sightings. In comparison, SINGs with ubiquitin or both ubiquitin and RPT-1 were observed. (*n* = 25 proximal oocytes). (JPG 242 kb)

Additional file 5: Figure S4. *skn-1* is not required for SING formation. Antibody staining was conducted on dissected gonads from *skn-1(zu129)* mutants or their heterozygous siblings. Worms were subjected to 500 mM NaCl for 60 min or 10 mM H_2_O_2_ for 30 min prior to dissection and staining. Gonads from heterozygous worms (7/100 oocytes) or *skn-1* mutants (7/100 oocytes) soaked in M9 showed no SINGs (examples not shown here). Heterozygous worms soaked in 500 mM NaCl (96/100 oocytes) or 10 mM H_2_O_2_ (83/100 oocytes) have SINGs as expected. *skn-1 (zu129)* worms soaked in 500 mM NaCl (78/100 oocytes) or 10 mM H_2_O_2_ (74/100 oocytes) also have SINGs. The merged image shows ubiquitin, proteasome and DAPI channels. A total of 100 oocytes were collected from 2 independent experiments for each condition (*n* = 20 worms). Scale bar indicates 10 μm. (JPG 697 kb)

Additional file 8: Figure S6. Lifespan of salt stressed worms. Lifespan studies were conducted on vector and *ubc-18* RNAi treated worms soaked in either M9 buffer or 500 mM NaCl for 60 min. No effects on the lifespan of the adult worms were detected. Data were collected from 3 independent experiments (*n* = 80 worms). (JPG 262 kb)

## Abbreviations

CHIP: C-terminus of Hsc70 Interacting Protein
FRAP: Fluorescence recovery after photobleaching
GFP: Green fluorescent protein
HSF1: Heat shock factor 1
mCh: mCherry
NF-κB: Nuclear factor kappa-light-chain-enhancer of activated B cells
NPP: Nuclear pore protein
PML: Promyelocytic leukemia
RNAi: RNA interference
SING: Stress induced nuclear granule
UPS: Ubiquitin proteasome system
