# Supplementary figures and images for: Stress induced nuclear granules form in response to accumulation of misfolded proteins in Caenorhabditis elegans
Source: BMC Cell Biol. 2017 Apr 19;18:18. doi: 10.1186/s12860-017-0136-x (PMC5395811; doi:10.1186/s12860-017-0136-x)

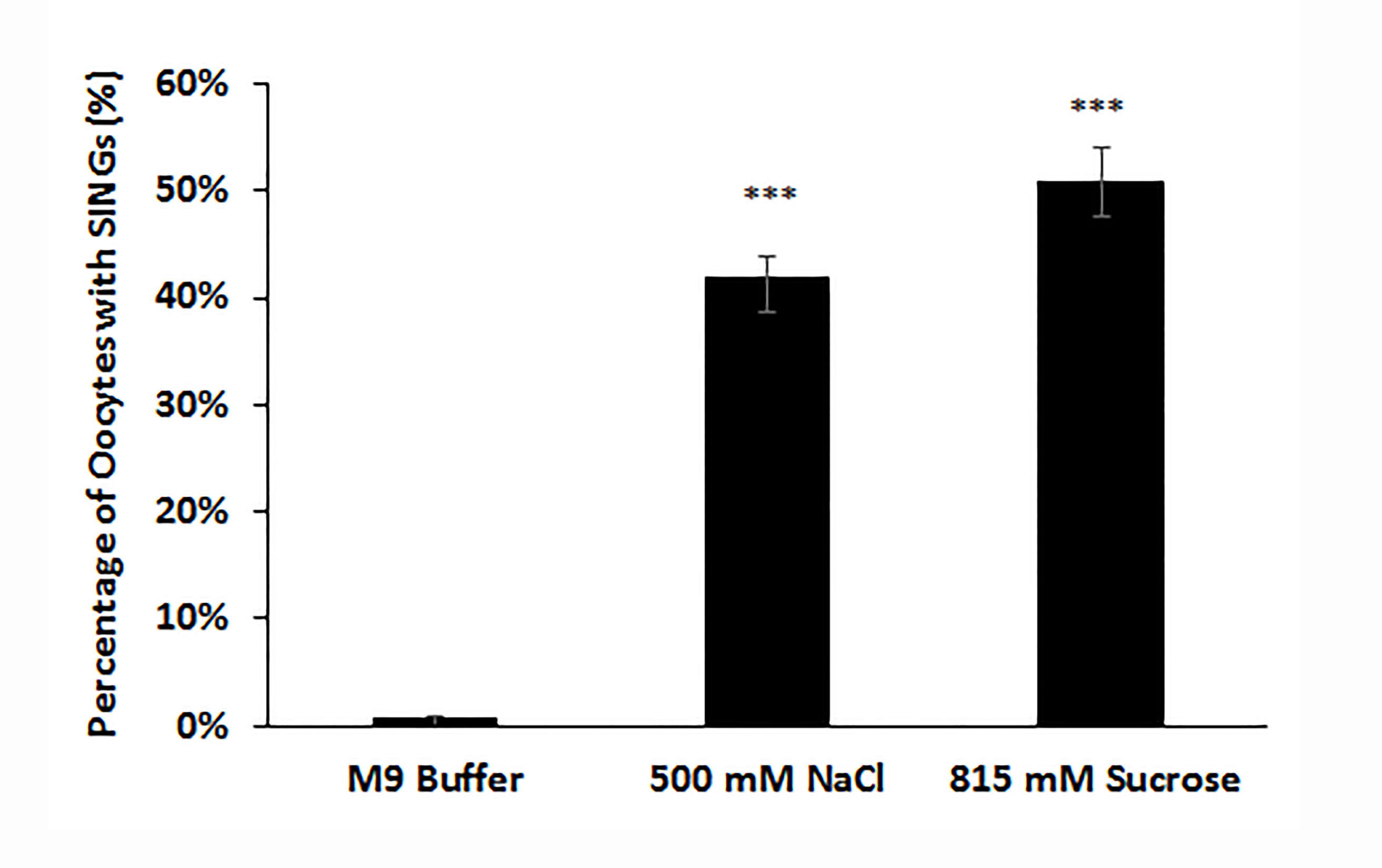

Supplement: Supplementary file 3 — High sucrose also induces SINGs. Young adult worms expressing GFP::Ub were soaked in either M9, 500 mM NaCl, or 815 mM sucrose for 1 h. Worms were then imaged on the confocal microscope and assessed for the presence of SINGs in oocyte nuclei. A higher concentration of sucrose was used due to the lower osmolarity of sucrose as compared to NaCl. For each condition, a total of 900 oocytes were observed from 3 independent experiments (n = 30 worms). Statistical significance was calculated by a Fisher’s Exact test: ***p < 0.001. (JPG 234 kb) [file 12860_2017_136_MOESM1_ESM.jpg]

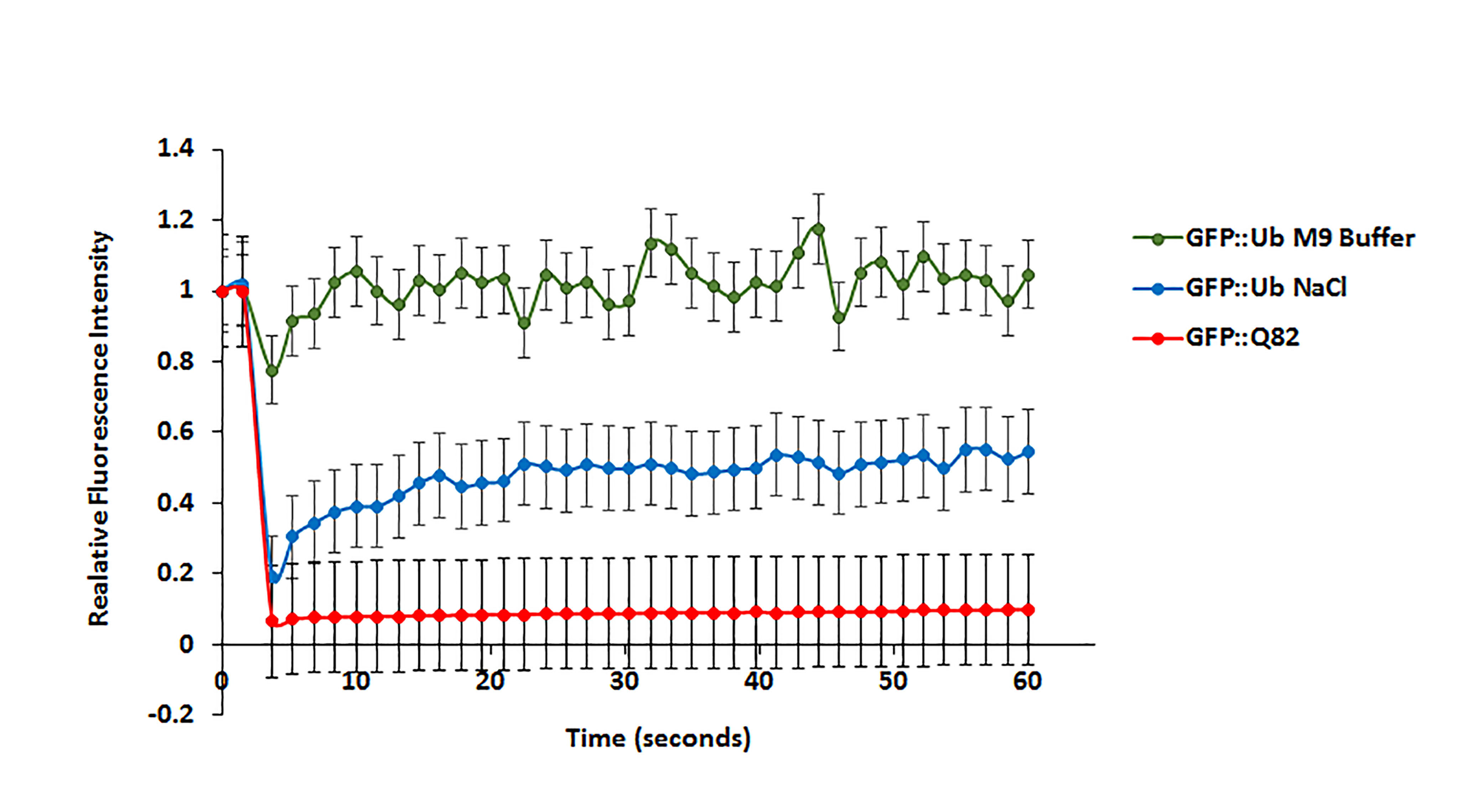

Supplement: Supplementary file 4 — FRAP analysis of SINGs. GFP::Ub in SINGs of the nuclei of salt stressed oocytes (blue) is compared to GFP::Ub in unstressed nuclei (green) and Q82::GFP in aggregates in muscle cells (red). The Q82::GFP shows little recovery of fluorescence over a one minute period, whereas, GFP::Ub in SINGs recovers to approximately 50% of initial level after 1 min. Graph shows the data from 10 individual FRAP experiments with standard errors indicated. (JPG 559 kb) [file 12860_2017_136_MOESM2_ESM.jpg]

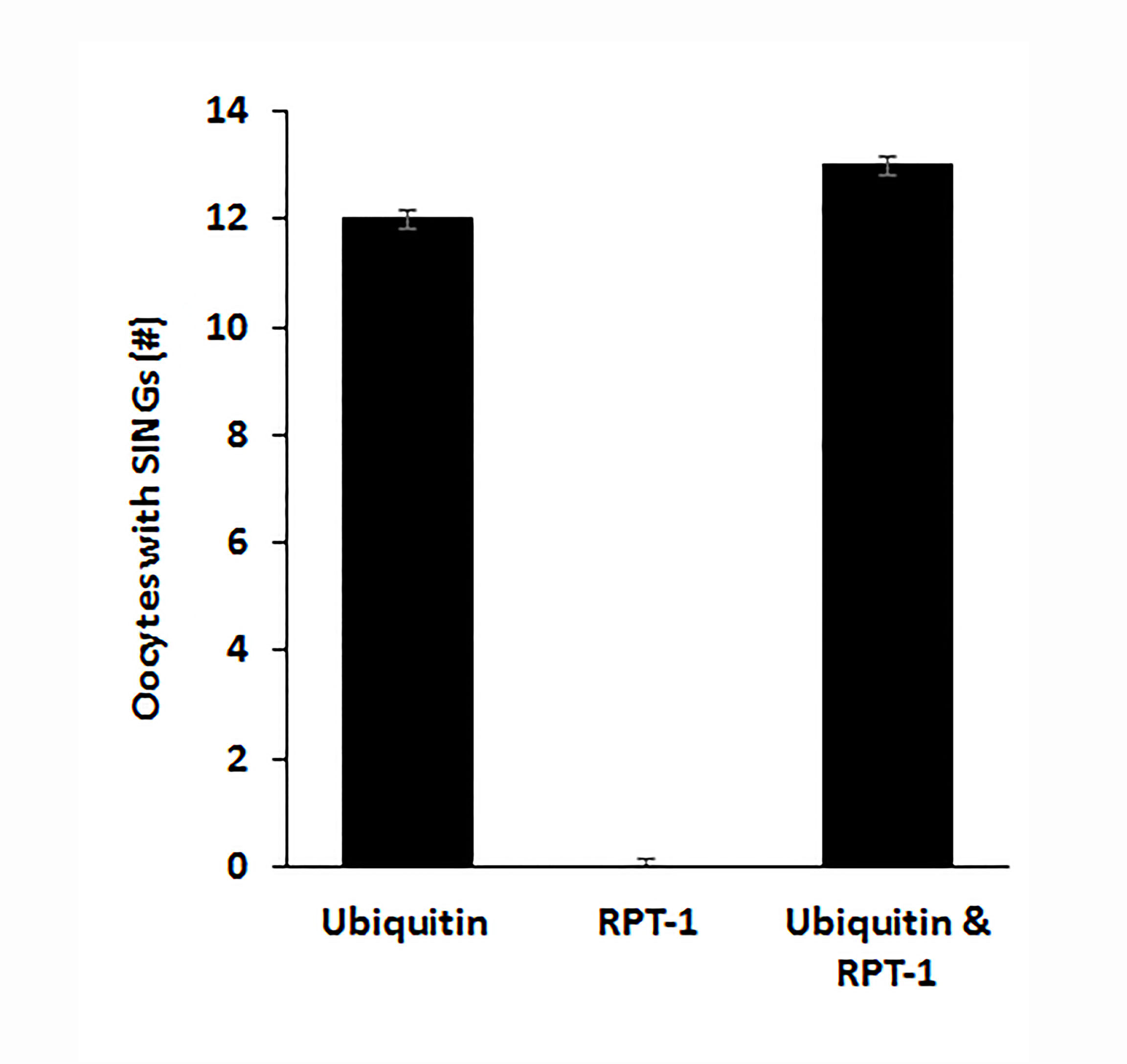

Supplement: Supplementary file 5 — Nuclear pore proteins, tubulin, PGL-1, and SMO-1 do not localize to SINGs during salt stress. (A) Worm strains expressing GFP::NPP were exposed to both M9 buffer and 500 mM NaCl for 60 min. The nuclear pore proteins responded to stress by occasionally forming concentrated areas of protein at the periphery of the nucleus: NPP-1 (19/250 oocytes) and NPP-7 (14/250 oocytes). A total of 250 oocytes were observed from 10 worms. (B) NPP-1 was crossed into a RPT-1::mCh expressing worm and then subjected to unstressed and salt stressed conditions. SINGs were not found to colocalize with NPP-1 (48/50 oocytes). A total of 50 proximal oocytes were observed from 10 worms. (C) Worms expressing GFP:: H2B and mCh:: smo-1 were soaked in M9 and 500 mM NaCl for 60 min. SMO-1 localized to the nucleolus in control and salt stress groups, but did not localize to SINGs (80/80 oocytes). A total of 80 proximal oocytes were observed from 2 independent experiments (n = 20 worms). (D) Tubulin and PGL-1 do not localize to SINGs during salt stress. GFP::tubulin worms were soaked in M9 buffer or 500 mM NaCl for 60 min and then observed under confocal microcopy. Both unstressed and stressed GFP::tubulin did not localize to SINGs (50/50 oocytes for each condition). A total of 50 oocytes were collected from 10 worms. Minor rearrangement of cytoplasmic tubulin was seen in stressed GFP::tubulin populations. GFP:: PGL-1 worms were soaked in M9 buffer or 500 mM NaCl for 60 min and then observed under confocal microcopy. Both unstressed and stressed GFP::PGL-1 did not localize to SINGs (240/240 oocytes for each condition). A total of 240 oocytes were collected from 6 worms. (E) Worms were stained with the RNA dye, SYTO 14. In unstressed worms high concentrations of RNA are detected in the cytoplasm and in the nucleolus (80/80 oocytes). In stressed worms, RNA localizes to cytoplasmic stress granules, but not to SINGs in the nucleus (80/80 oocytes). A total of 80 oocytes were collected from two independent [file 12860_2017_136_MOESM3_ESM.jpg]

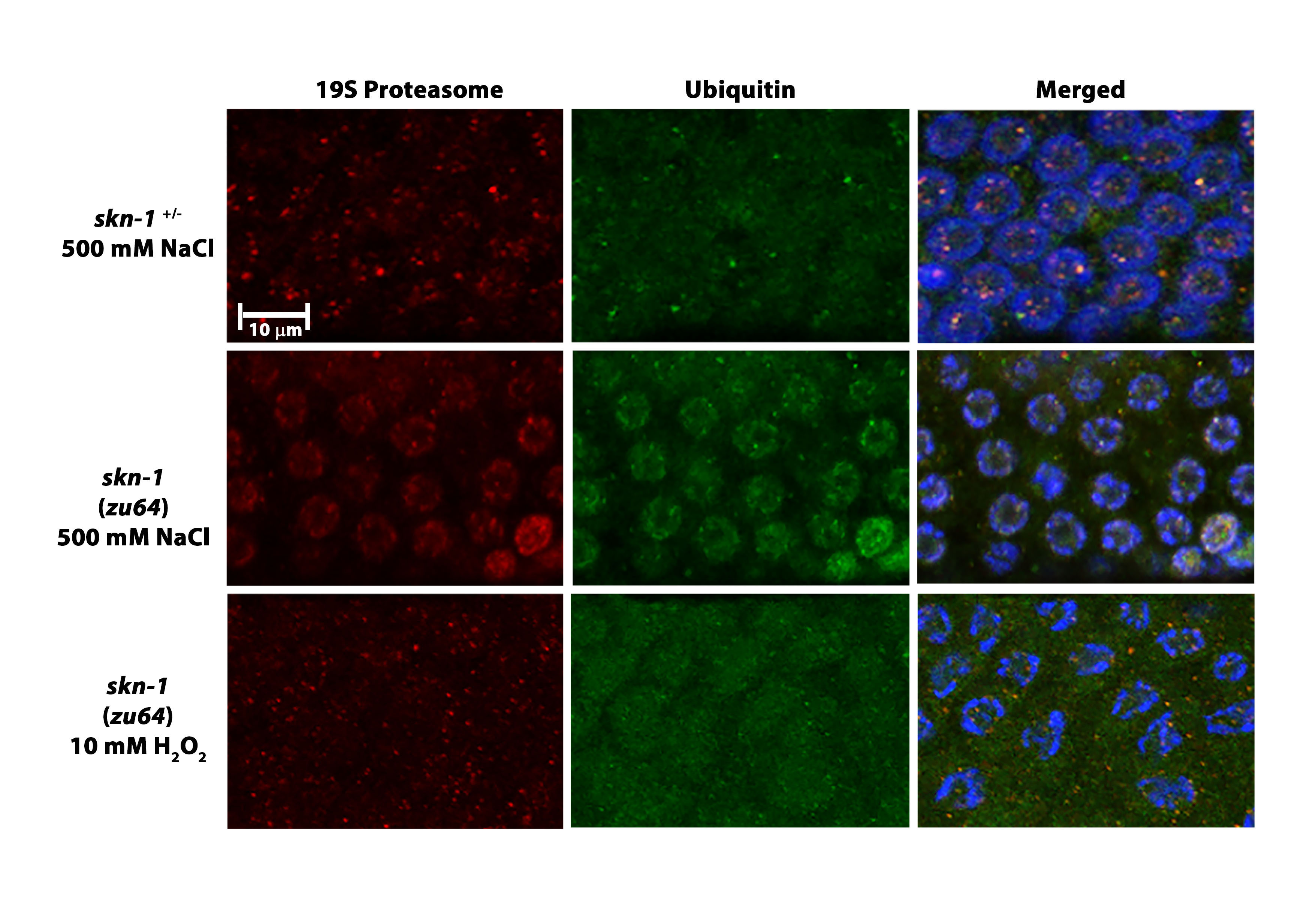

Supplement: Supplementary file 6 — Time-lapse analysis of SINGs. Worms expressing GFP::Ub (green) and RPT-1::mCh (red) and soaked in 500 mM NaCl for 60 min were imaged for 30 min with an image taken every minute. When SINGs first appeared in the time-lapse series, they were assessed for the presence ubiquitin, RPT-1, or both ubiquitin and RPT-1. The numbers for each category are shown in the graph. RPT-1 alone was not observed at any of the initial SING sightings. In comparison, SINGs with ubiquitin or both ubiquitin and RPT-1 were observed. (n = 25 proximal oocytes). (JPG 242 kb) [file 12860_2017_136_MOESM4_ESM.jpg]

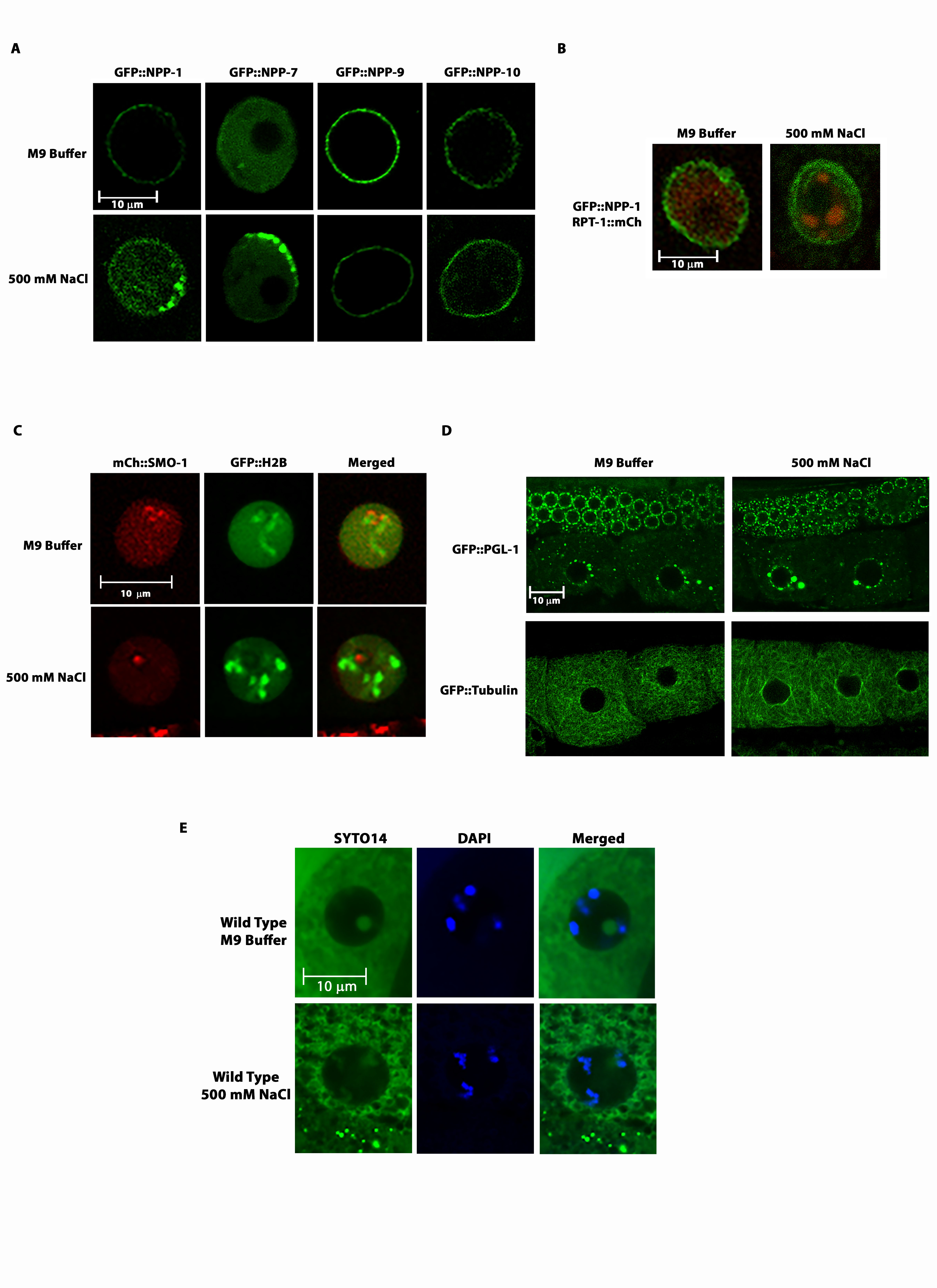

Supplement: Supplementary file 7 — skn-1 is not required for SING formation. Antibody staining was conducted on dissected gonads from skn-1(zu129) mutants or their heterozygous siblings. Worms were subjected to 500 mM NaCl for 60 min or 10 mM H2O2 for 30 min prior to dissection and staining. Gonads from heterozygous worms (7/100 oocytes) or skn-1 mutants (7/100 oocytes) soaked in M9 showed no SINGs (examples not shown here). Heterozygous worms soaked in 500 mM NaCl (96/100 oocytes) or 10 mM H2O2 (83/100 oocytes) have SINGs as expected. skn-1 (zu129) worms soaked in 500 mM NaCl (78/100 oocytes) or 10 mM H2O2 (74/100 oocytes) also have SINGs. The merged image shows ubiquitin, proteasome and DAPI channels. A total of 100 oocytes were collected from 2 independent experiments for each condition (n = 20 worms). Scale bar indicates 10 μm. (JPG 697 kb) [file 12860_2017_136_MOESM5_ESM.jpg]

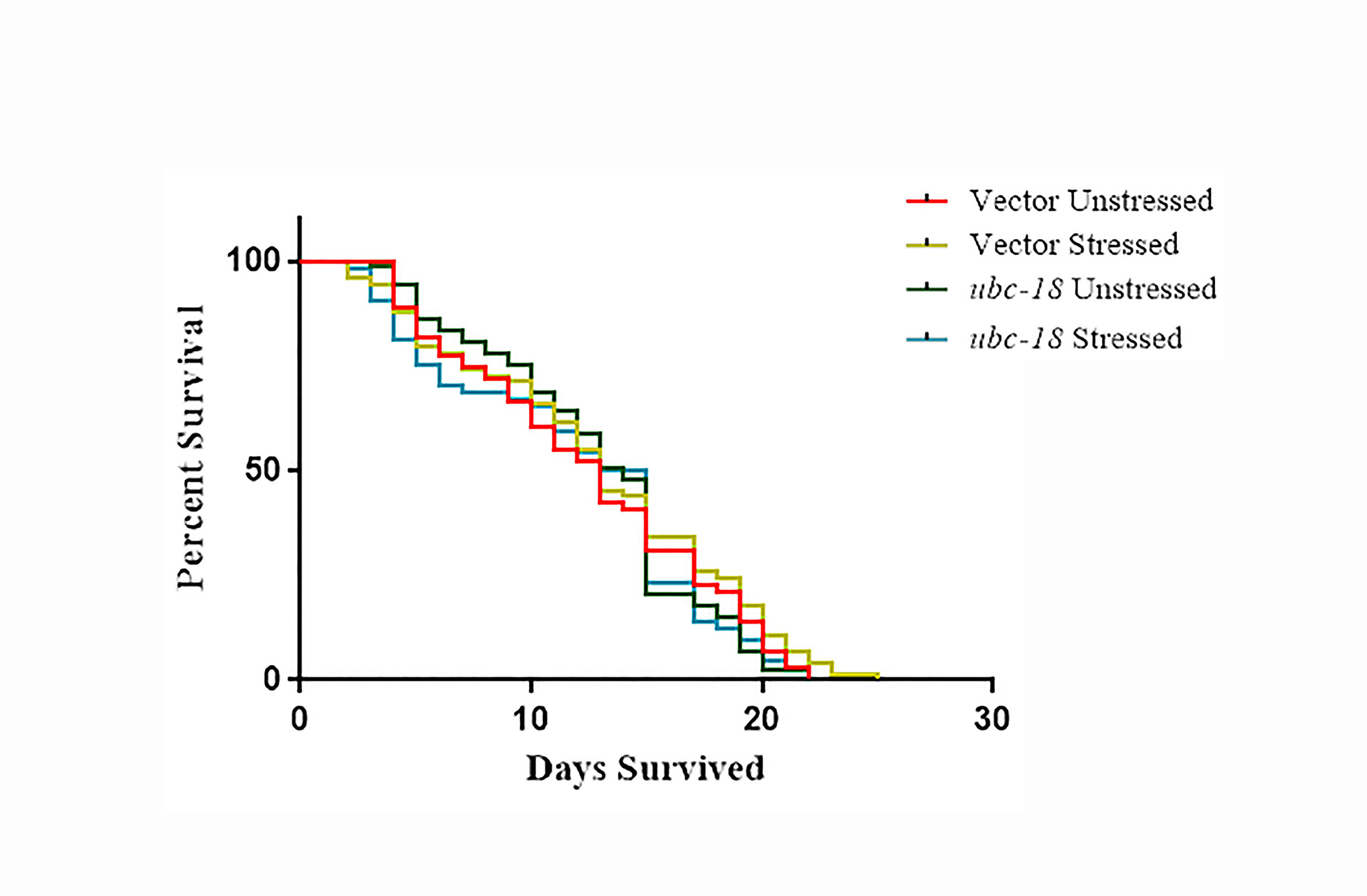

Supplement: Supplementary file 8 — Lifespan of salt stressed worms. Lifespan studies were conducted on vector and ubc-18 RNAi treated worms soaked in either M9 buffer or 500 mM NaCl for 60 min. No effects on the lifespan of the adult worms were detected. Data were collected from 3 independent experiments (n = 80 worms). (JPG 262 kb) [file 12860_2017_136_MOESM8_ESM.jpg]
